# Seagrass ecosystem multifunctionality under the rise of a flagship marine megaherbivore

**DOI:** 10.1111/gcb.16464

**Published:** 2022-11-04

**Authors:** Marjolijn J. A. Christianen, Fee O. H. Smulders, Jan Arie Vonk, Leontine E. Becking, Tjeerd J. Bouma, Sabine M. Engel, Rebecca K. James, Mabel I. Nava, Jaco C. de Smit, Jurjan P. van der Zee, Per J. Palsbøll, Elisabeth S. Bakker

**Affiliations:** ^1^ Aquatic Ecology and Water Quality Management Group Wageningen University & Research Wageningen The Netherlands; ^2^ Marine Evolution and Conservation Group Groningen Institute for Evolutionary Life Sciences, University of Groningen Groningen The Netherlands; ^3^ Department of Freshwater and Marine Ecology Institute for Biodiversity and Ecosystem Dynamics (IBED), University of Amsterdam Amsterdam The Netherlands; ^4^ Aquaculture and Fisheries group Wageningen University & Research Centre Wageningen The Netherlands; ^5^ Department of Estuarine and Delta Systems, Royal Netherlands Institute for Sea Research (NIOZ) Yerseke The Netherlands; ^6^ Department of Physical Geography, Faculty of Geosciences Utrecht University Utrecht The Netherlands; ^7^ STINAPA, Bonaire National Parks Foundation Bonaire Caribbean Netherlands; ^8^ Biogeochemistry and Modeling of the Earth System Group Université libre de Bruxelles Bruxelles Belgium; ^9^ Sea Turtle Conservation Bonaire Bonaire Caribbean Netherlands; ^10^ Center for Coastal Studies Provincetown Massachusetts USA; ^11^ Department of Aquatic Ecology, Netherlands Institute of Ecology (NIOO‐KNAW) Wageningen The Netherlands; ^12^ Wildlife Ecology and Conservation Group, Wageningen University & Research Wageningen The Netherlands

**Keywords:** *Chelonia mydas*, defaunation, ecosystem multifunctionality index, ecosystem services, megaherbivore recovery, nonlinear thresholds, resilience, *Thalassia*, trophic cascade, tropical seagrass

## Abstract

Large grazers (megaherbivores) have a profound impact on ecosystem functioning. However, how ecosystem multifunctionality is affected by changes in megaherbivore populations remains poorly understood. Understanding the total impact on ecosystem multifunctionality requires an integrative ecosystem approach, which is especially challenging to obtain in marine systems. We assessed the effects of experimentally simulated grazing intensity scenarios on ecosystem functions and multifunctionality in a tropical Caribbean seagrass ecosystem. As a model, we selected a key marine megaherbivore, the green turtle, whose ecological role is rapidly unfolding in numerous foraging areas where populations are recovering through conservation after centuries of decline, with an increase in recorded overgrazing episodes. To quantify the effects, we employed a novel integrated index of seagrass ecosystem multifunctionality based upon multiple, well‐recognized measures of seagrass ecosystem functions that reflect ecosystem services. Experiments revealed that intermediate turtle grazing resulted in the highest rates of nutrient cycling and carbon storage, while sediment stabilization, decomposition rates, epifauna richness, and fish biomass are highest in the absence of turtle grazing. In contrast, intense grazing resulted in disproportionally large effects on ecosystem functions and a collapse of multifunctionality. These results imply that (i) the return of a megaherbivore can exert strong effects on coastal ecosystem functions and multifunctionality, (ii) conservation efforts that are skewed toward megaherbivores, but ignore their key drivers like predators or habitat, will likely result in overgrazing‐induced loss of multifunctionality, and (iii) the multifunctionality index shows great potential as a quantitative tool to assess ecosystem performance. Considerable and rapid alterations in megaherbivore abundance (both through extinction and conservation) cause an imbalance in ecosystem functioning and substantially alter or even compromise ecosystem services that help to negate global change effects. An integrative ecosystem approach in environmental management is urgently required to protect and enhance ecosystem multifunctionality.

## Graphical abstract

Three scenarios of megaherbivore grazing intensity can be observed in tropical seagrass ecosystems with green turtles as megaherbivores across the world (Figure a). The turtle's ecological role is rapidly unfolding in numerous foraging areas where populations are recovering through conservation after centuries of decline, with an increase in recorded overgrazing episodes. In field experiments, researchers assessed the effects of simulated grazing intensity scenarios on ecosystem functions and multifunctionality (Figure b) in a tropical Caribbean seagrass ecosystem over an 18‐month period.

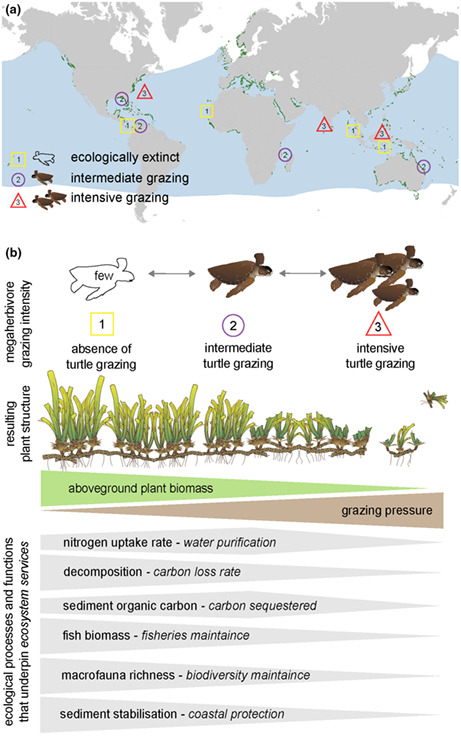

## 1 INTRODUCTION

Humans rely on a multitude of services provided by Earth's ecosystems, such as food, water, and protection as well as climate buffering (Costanza et al., 2014; Millenium Ecosystem Assessment, 2005). However, humans are greatly impacting megafauna population numbers, both through overexploiting and degrading entire ecosystems and their fauna (WWF, 2020; Rockström et al., 2009) and also through successful conservation and restoration efforts that allow some populations to rebound locally (Lotze et al., 2011; Warren, 2011). These changes in megafauna populations can induce large‐scale changes in terrestrial, freshwater, and marine ecosystems, which in turn impairs ecosystem functions and services, as found across ecosystems and biogeographic zones, including tundra, savanna, and rainforests (Dirzo et al., 2014; Doughty et al., 2016; Estes et al., 2011; Galetti et al., 2015; McCauley et al., 2015; Zimov & Zimov, 2014). Additionally, changes to one species can also disrupt the complex equilibrium between trophic levels if predators and their prey are impacted in a different way (e.g., large herbivore recovery in a system where their food source is still in decline) or recovering at different time scales (Duarte et al., 2020). This potential has been illustrated in several classic studies of cascading, top‐down effects triggered by megafaunal defaunation through extirpation of sharks, otters, and cetaceans (Ainley et al., 2006; Baum & Worm, 2009; Estes et al., 2009; Estes & Palmisano, 1974; Heithaus, Frid, et al., 2008; Steneck & Sala, 2005).

A decrease or increase in marine megafauna populations coincides with changes in key ecosystem functions and services, such as coastal erosion protection (Coverdale et al., 2014), nutrient transport (Doughty et al., 2016), carbon sequestration (Wilmers et al., 2012), and ecosystem resilience (Hughes et al., 2016; Steneck & Sala, 2005). However, extrapolating results from a single function to infer the role of marine megafauna in complex systems ignores the interplay among functions, as well as our desire to simultaneously extract multiple goods and services from high‐functioning ecosystems. To solve this we need an integrative assessment of the effects of changing megafauna abundance on the entire ecosystem, its functions and services and the interplay among functions, termed ecosystem multifunctionality (Byrnes et al., 2014; Hensel & Silliman, 2013), which is currently lacking. Furthermore, there is no evidence of causation, as experimental support for the ecosystem impacts of changes of marine megafaunal on multifunctionality remains absent so far because experimental support for such integrative assessment is challenging to obtain, especially in marine systems.

**FIGURE 1:**
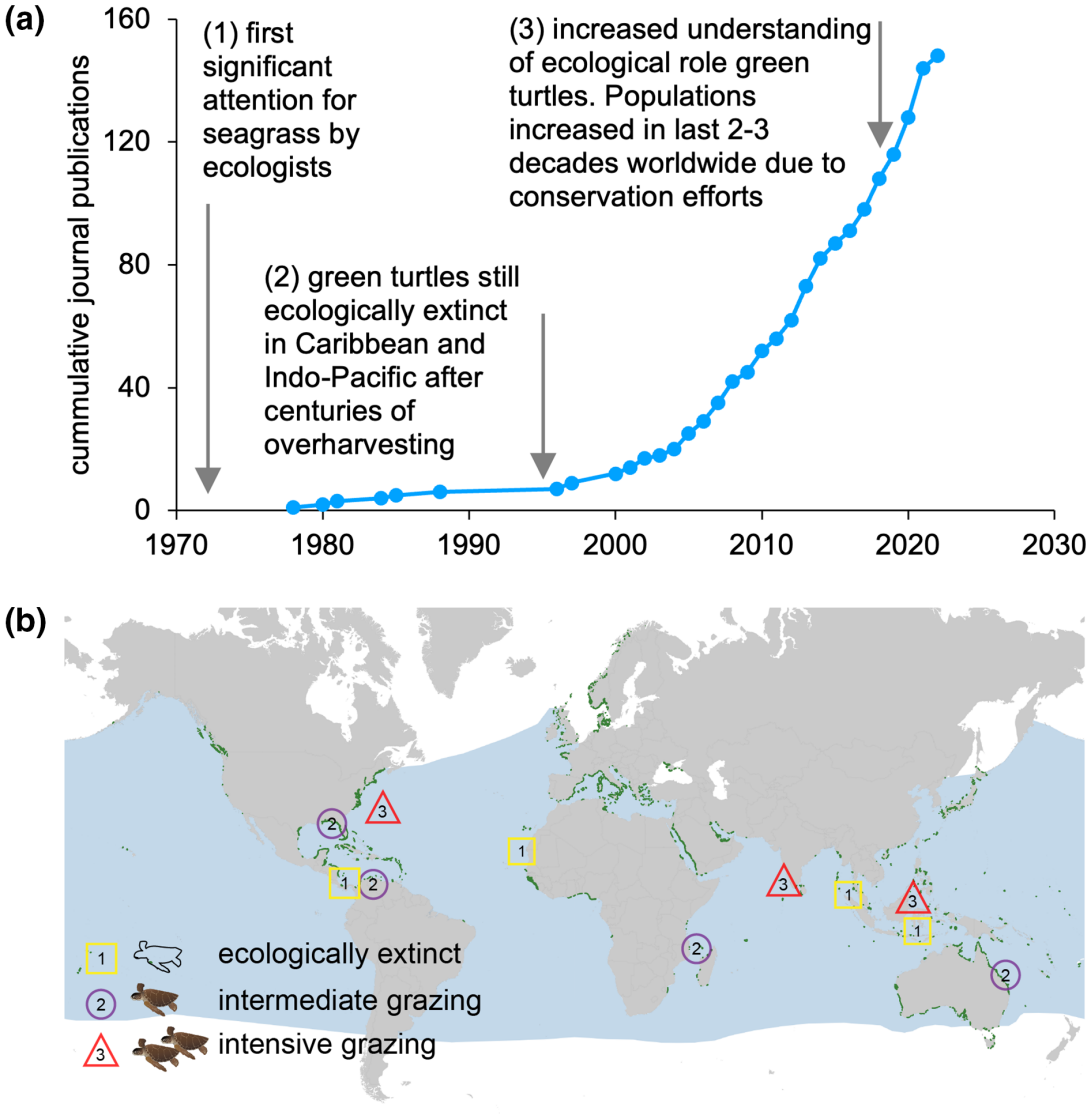
(a) The number of publications on seagrass and green turtle grazing in peer‐reviewed journals is accelerating over time (Web of Science, Scopus, Google Scholar 1960–2022, Supplementary information text S1) mirroring the recovery of green turtle populations. Arrow 1: (McRoy & Helfferich, 1977; Thayer et al., 1977); arrow 2: (Jackson, 1997), arrow 3: (Chaloupka et al., 2008; Mazaris et al., 2017; Weber et al., 2014). (b) A selection of sites illustrates that all three different grazing scenarios for green turtles occur in coastal (sub‐)tropical seagrass ecosystems around the world, in all three ocean basins where green turtles are found. Green dots: global seagrass distribution (UNEP‐WCMC & Short, 2021), blue: distribution of the green turtle, *Chelonia mydas*, (Kot et al., 2022). (Scenario 1) (Gaubert‐Boussarie et al., 2021; Jackson, 1997; Jones et al., 2018; van der Laan & Wolff, 2006; Vonk et al., 2008); (Scenario 2) (Ballorain et al., 2010; Christianen et al., 2019; Gulick et al., 2020; Molina Hernández & van Tussenbroek, 2014; Rodriguez & Heck, 2020; Scott et al., 2020); (Scenario 3) (Christianen et al., 2014; Fourqurean et al., 2019; Gangal et al., 2021).

Here, we assessed the effects of changing megaherbivore populations on ecosystem multifunctionality in a tropical seagrass ecosystem. Undisturbed seagrass ecosystems are hotspots for marine megafauna including sea turtles, sharks, dugongs, dolphins, otters, and crocodiles (Sievers et al., 2019), and provide crucial ecosystem services (Nordlund et al., 2018). Seagrasses evolved under grazing pressure by mammalian megaherbivores (sea cows or Sirenians such as dugongs and manatees) and by its dominant megaherbivore, the green turtle (*Chelonia mydas)* (Aragones & Marsh, 2000; Domning, 2001) and thus, grazed seagrass meadows presumably represent the “natural” state of seagrass ecosystems (Christianen et al., 2021) until overexploitation began centuries ago (Jackson et al., 2001; Thayer et al., 1984). After the decimation of turtle populations, roughly between 1800 and 1990, long before modern ecological investigations began, seagrass meadows were left composed of large, slow‐growing climax species with high seagrass biomass (Jackson, 1997). Since successful conservation measures to protect nesting areas and international law prohibiting turtle trade were established, an increasing number of seagrass meadows is experiencing a rise in green turtle populations (Chaloupka et al., 2008; Mazaris et al., 2017; Weber et al., 2014). As a result, more and more seagrass meadows are recovering to their natural grazed state in the last decade. This is reflected by acceleration on the number of publications on seagrass, megaherbivores, and turtles in peer‐reviewed journals over time (Figure 1a; Supplementary information text S1). Turtle population growth may be enhanced further by the absence of their main predator (Tiger shark, *Galeocerdo cuvier*) due to shark overfishing (Heithaus et al., 2014) and by the loss of seagrass habitat due to other anthropogenic stressors, which stimulate turtle densities to increase in the remaining habitat (Christianen et al., 2014). This has resulted in an increase in reports of turtles overgrazing the seagrass, that is, when grazing rates exceed production rates (Christianen et al., 2014; Fourqurean et al., 2019; Gangal et al., 2021; Williams, 1988) in some cases leading to seagrass collapse (Christianen et al., 2014; Gangal et al., 2021). The intensity of megaherbivore grazing can thus determine the seagrass biomass, shoot density, and canopy structure (Burkholder et al., 2013; Nowicki et al., 2018; Scott et al., 2018; Smulders et al., 2022), ranging from low seagrass biomass when sea turtles are abundant, to high seagrass biomass when turtles are absent (Figure 3), which may affect ecosystem multifunctionality.

We translated these observations into our aim of assessing the impact of increasing megaherbivore densities on key seagrass ecosystem functions and multifunctionality. This was tested in an experimental design in which the seagrass was excluded from turtle grazing (representing absence of turtles), exposed to intermediate turtle grazing (representing the naturally grazed scenario), and lastly, we manipulated the seagrass to mimic a scenario of intensive grazing or overgrazing by turtles by removing plant biomass, based on literature showing this mechanism (Fourqurean et al., 2019; Gangal et al., 2021). After 18 months of experimentation, we measured seven ecosystem functions and captured the overall effects in a novel, integrated seagrass ecosystem multifunctionality index.

## 2 MATERIALS AND METHODS

### 2.1 Study system

The experiment was conducted in a tropical, subtidal seagrass meadow, located within Lac Bay, Bonaire, Caribbean Netherlands (12°06′N 068°14′W). Lac Bay contains ~200 ha of seagrass and is designated to be a wetland of international importance under the Ramsar Convention. Seagrass meadows were mainly dominated by the native seagrass, *Thalassia testudinum*, and to a lesser extent *Syringodium filiforme*, as well as the invasive seagrass, *Halophila stipulacea* (Christianen et al., 2019) along with beds of the calcareous algae *Halimeda* spp. Today, Lac Bay's seagrass meadows are home to one of the largest green turtle foraging aggregations in the southern Caribbean (Debrot et al., 2012; Rivera‐Milán et al., 2019). The abundance of green turtles (*Chelonia mydas*) has been recovering in the leeward Dutch Caribbean islands in recent decades after past depletion due to overharvesting (Jackson, 1997). By contrast, overexploited top predators that feed on green turtles, such as tiger sharks have not shown any evidence of recovery in the Caribbean (Ward‐Paige et al., 2010), with only occasional observations on Bonaire. The green turtle population in Lac Bay grazed only on seagrass leaves with plenty of seagrass biomass still present. The seagrass meadow can withstand the current grazing pressure and remains a high productivity (Christianen et al., 2019). Hence, we classified the current meadow as being exerted to intermediate grazing pressure. Other foraging areas with very high green turtle abundances are subject to much higher grazing intensities than observed in Lac Bay (Christianen et al., 2014, 2021; Gangal et al., 2021). Other megaherbivores like manatees (*Trichechus manatus*) also feed on seagrass. However, while manatees were abundant in the Southern Caribbean region before the European colonization during the 17th–19th centuries, they were still absent from the region at the time of the study (Debrot et al., 2013; Jackson, 1997). Mesoherbivore fish were abundant in the bay but only in shallower areas (e.g., the mangrove fringe) where the absence of turtle grazing leads to a high canopy that provides food and shelter to a high diversity of fish (Smulders et al., 2022).

### 2.2 Approach and megaherbivore grazing intensity treatments

To assess the impact of megaherbivore grazing intensity on ecosystem multifunctionality, we have experimentally manipulated seagrass biomass and grazing intensity to simulate three progressing grazing intensity scenarios, all of which can be found in three ocean basins (Figure 1b): (1) no turtle grazing, representing the absence of turtles. The absence of turtle grazing (or intensive grazing by smaller herbivores) results in high seagrass biomass as observed in many current modern seagrass meadows where turtles remain ecological extinct (Gaubert‐Boussarie et al., 2021; Jackson, 1997; Jones et al., 2018; van der Laan & Wolff, 2006; e.g., Vonk et al., 2010); (2) intermediate turtle grazing, representing presence by turtles. Ecosystems were exposed to natural or intermediate grazing intensity resulting in intermediate seagrass biomass, with plenty of leaf biomass still present as observed in meadows with turtles (Christianen et al., 2019; Molina Hernández & van Tussenbroek, 2014); (3) intensive turtle grazing, representing the accumulation of turtles, resulting in very high grazing pressure and sometimes “overgrazing,” that is, when grazing rates exceed production rates, and very low seagrass biomass as observed in areas with turtle accumulation (Fourqurean et al., 2019; Gangal et al., 2021).

We manipulated the seagrass biomass corresponding with the three different grazing intensity treatments with a combination of exclosure and seagrass removal treatments. In Treatment 1, turtle grazing was excluded from the plots by using underwater cages (1.5 m × 1.5 m × 0.5 m with walls of galvanized 9 mm steel wires and 15 cm mesh size). The cages excluded sea turtles but permitted the movement of small‐bodied animals (e.g., fish), did not attract additional fish and did not inhibit light transmission to the seagrass bed (Christianen et al., 2012). The vertical walls of each cage were extended 30 cm into the sediment to prevent subterranean movement and intrusion of large animals. Algae growth on the cages was minimal during the experiment as it was checked every 2 weeks and removed when necessary. For Treatment 2, the plots were left exposed to intermediate green turtle leaf grazing. Each plot was marked by four galvanized steel pins protruding 10 cm above the sediment and not subjected to any changes. Turtle grazing was constant over the 18 months. In Treatment 3, plots were exposed to high‐intensity grazing. Here, all above‐ and belowground seagrass biomass was removed from the plot at the start of the experiment (July 2015) to mimic the effects of high‐intensity turtle grazing, observed in areas with a high abundance of sea turtles that induced a shift from *Thalassia* to bare sand (Gangal et al., 2021) or excavated roots (Christianen et al., 2014). The excavating behavior is atypical of the cultivated grazing behavior that has been widely documented in the literature for Caribbean meadows (Bjorndal, 1980; Gulick et al., 2020; Ogden et al., 1983), where turtle densities and seagrass species numbers and grazing intensity are typically lower than tropical meadows elsewhere. However, if turtles only intensively graze on aboveground leaves, this can also lead to bare patches as *Thalassia* meadows become depleted and can no longer recover. This has been observed in the Pacific ocean within 5 years after the arrival of dense turtle aggregations (Gangal et al., 2021). Under continued turtle accumulation, the transition of Thalassia to bare patches is likely to arise elsewhere and has already been observed in Bermuda (Government of Bermuda, 2021), Bonaire (Pers. obs. MJAC and FOHS), and the Bahamas (Smulders et al., 2022). Plots were marked as in Treatment 2, thereby permitting re‐colonization by clonal expansion of surrounding seagrass during the experiment while still being exposed to intermediate turtle grazing.

### 2.3 Experiment

The experiment was conducted over a period of 18 months (from July 2015 to February 2017). Fifteen plots (1.5 m × 1.5 m) were selected at similar water depths (2.0 m ± 0.3 m), similar plant biomass, and cover and were deployed over an area of 500 m^2^. The three treatments were applied randomly to the chosen plots to avoid potentially confounding effects of small‐scale spatial heterogeneity.

The resulting seagrass biomass was quantified at the start of the experiment (to ensure plots had similar biomass) and at the end of the experiment in all experimental plots from a core sample (15.3 cm diameter, 20 cm depth) collected at the center of each plot, together with leaf productivity, shoot density, and canopy height. Aboveground plant parts (leaves and sheaths) were separated from belowground parts (roots and rhizomes) before processing and analysis. Aboveground parts were rinsed with water to remove epiphytes and sediment as well as other attached materials. After drying (48 h at 60°C) the aboveground biomass of *T. testudinum* in each core was quantified as the combined dry weight (DW) of *T. testudinum* leaves and sheaths.

### 2.4 Ecosystem functions

We measured seven variables serving as proxies for ecosystem functions and processes underpinning essential seagrass ecosystem services (Table 1, Barbier et al., 2011; Nordlund et al., 2018): nutrient cycling, decomposition rates, carbon storage, fish biomass, macroinvertebrate species richness (α diversity), sediment stability, and resilience to invasive species. For five processes, the corresponding variables were measured within each plot of the treatments (carbon content, decomposition rate, nutrient cycling, macroinvertebrate species richness, and percentage of invasive seagrass). Fish biomass was estimated after cages were removed to avoid cage effects. Sediment stabilization was estimated in the close vicinity of the experimental plots, in selected plots where biomass measurements confirmed similar aboveground biomass, as the experimental plots contained an insufficient area of undisturbed sediment.

**TABLE 1:** Methods used to measure seven proxies of seagrass ecosystem services and functions

Ecosystem service	Method	Reference
*Ecosystem process and function*	*Proxy measured herein (Sampling timing)*	
Water purification Nutrient cycling	1. *Net leaf nitrogen uptake rate*, *calculated as* seagrass leaf production (using plastochrone method) × leaf nitrogen content, measured using elemental analyzer. (*S*, *E)*	Christianen et al. (2019)/Short and Duarte (2001))
Carbon sequestration Biochemical activity	2. *Decomposition rate*, *determined from the* Tea bag index (*over last 61 days of experiment)*	Keuskamp et al. (2013)
	3. *Sediment organic carbon content* using dry combustion method with the elemental analyzer *(E)*	Howard et al. (2014)
Fisheries maintenance Provisioning of habitat, shelter, nursery	4. *Fish biomass* using stationary‐point‐count‐method and SCUBA. Biomass estimated using species specific weight–length relationships *(E)*	Polunin and Roberts (1993)
	5. *Macrofauna (invertebrate) richness* from sediment cores and net sweeps *(E)*	Vonk et al. (2010)
Coastal protection and erosion control Wave attenuation and sediment stabilization	6. *Sediment stabilization*, measured as threshold shear velocity, from unilateral field flume measurements. *(E)*	James et al. (2020)
Tourism, research Maintaining wildlife habitat	7. Invasive species buffering assessed as the area cover of invasive species *Halophila stipulacea*. A non‐preferred species for megafauna. *(E)*	Smulders et al. (2017)

*Note*: Ecosystem services reflect benefits (of monetary value) provided to humanity and are underpinned by examples of ecosystem processes and functions, adapted from Barbier et al. (2011). Sampling timing and frequency are given between brackets. S: start of the experiment, E: end of the experimental period. The proxy measured herein outlines the actual variant(s) of those processes and functions that we quantified.

#### 2.4.1 Estimation of nutrient cycling

Nutrient cycling was assessed using net aboveground seagrass nitrogen uptake as a proxy and was estimated by multiplying leaf productivity with leaf nitrogen content. Seagrass productivity was assessed using the plastochrone method (Short & Duarte, 2001) and the dry weight of new regrowth was measured (48 h at 60°C) after an 11‐day interval at the start and end of the experimental period. Leaf nitrogen content was estimated from the material used to quantify seagrass aboveground biomass. Dried leaves were ground and subsequently analyzed using an elemental analyzer coupled as described in Christianen et al. (2019).

#### 2.4.2 Estimation of decomposition rates

Organic matter decomposition rates were quantified using the “tea bag” index (Keuskamp et al., 2013). The approach employs commercially available tea bags as a standardized assessment. Five tea bags of two types of tea with different characteristics (rooibos tea, Lipton Inc., EAN: 8722700 18843 8, and green tea, Lipton Inc., EAN: 8722700 05552 5) were buried at 8 cm depth in each plot. The tea bags were deployed during the last 2 months of the field treatments and recovered after 61 days. Soil particles were removed and the tea and bags were dried (48 h at 60°C) and weighed. The use of tea types with contrasting decomposability served as the basis for the estimation of a decomposition curve from a single temporal sample. The decomposition rate (*k*) was calculated as described by Keuskamp et al. (2013), using a hydrolyzable fraction of 0.552 g g^−1^ and 0.842 g g^−1^ for rooibos tea and green tea, respectively.

#### 2.4.3 Estimation of carbon storage

*S*ediment organic carbon storage was estimated as the percentage of carbon in the sediment. Small sediment cores (22.9 mm diameter, 50 mm depth, yielding a sediment volume of 20.6 cm^3^) were collected from the cores used to extract aboveground plant biomass. Sediment samples were dried (48 h at 60°C) and weighed to determine the dry bulk density (DBD; mg DW m^−3^). Corrections for inorganic carbon (i.e., calcium carbonate, CaCO_3_) were undertaken on subsamples that were incinerated (4 h at 500°C) and the resulting ash (containing the inorganic carbon) was weighed. The percentage of carbon in sediment and ash was measured using a Thermo Scientific™ Delta V isotope ratio mass spectrometer coupled with a Thermo Scientific Interscience “Flash” Elemental Analyzer™, series 112 (Thermo Scientific Inc.). Standards were included in every five samples using ISE 946 reference material and the certified calibration standard Acetanilide (OAS certificate 293514). The percentage of inorganic carbon in the ash was subtracted from the estimate of total carbon in the sediment to obtain the percentage of organic carbon in the sediment (Howard et al., 2014).

#### 2.4.4 Estimates of fish biomass and macroinvertebrate species richness

Fish biomass and macroinvertebrate species richness were assessed from visual underwater census, stationary point‐count‐methods (Dorenbosch et al., 2005; Polunin & Roberts, 1993), and sediment core samples. Fish biomass was assessed in a quadrat of 1.5 m × 1.5 m. Counts were conducted after a wait time of 5 min to minimize disturbance. During the first 7 (of a total of 10) min of observation time, fish species identification and counting were conducted from outside the sampling quadrat. During the last 3 min, the observer moved through the quadrat to identify and count smaller fish hiding within the canopy. All macrobenthic invertebrates (epifauna >1 cm) were identified and counted inside the quadrat during these last 3 min (Vonk et al., 2010). Additionally, infauna was collected and counted from the sediment cores collected for plant biomass analysis (see above) after sieving the sediment (1 mm round mesh). The collected macrobenthic invertebrates were identified to as taxonomic class. Because species richness of both in‐ and epifauna was highly correlated (Figure S2, *R* ^2^ .72, *p* < .001) the data of in‐ and epifauna species richness were combined for each plot and reported per unit area (m^2^).

All fish records were classified into 2.5 cm size classes and used to estimate total fish biomass. Estimation of size classes was trained by repeatedly estimating the sizes of objects placed underwater representing all size classes until the observer was able to determine length with a maximum deviation of 2.5 cm for objects less than 20 cm long (Humann & DeLoach, 1989). Fish biomass was estimated from the size estimates for each species using species‐specific weight–length relationships (WLR), defined as *W = a* × *L* ^*b*^, where *W* is fish total dry weight in grams, *L* is the length in cm, *a* is a species‐specific coefficient that relates to body shape and *b* is the exponent relating to species‐specific growth form (Bouchon‐Navaro et al., 2006; Froese et al., 2014). The parameter estimates for *a* and *b* were obtained from previously published data (Bouchon‐Navaro et al., 2006) based on 50 different fish species collected from seagrass meadows in the Lesser Antilles.

#### 2.4.5 Estimation of sediment stability

Sediment stability was quantified by measuring the threshold flow velocity, that is, the velocity of water at which sediment was mobile, in a portable “unidirectional‐flow flume,” the TiDyFLOW flume (James et al., 2019). The portable flume was placed nearby the experimental plots on plots with seagrass biomass comparable to the treatments. Measurements were conducted at three plots for each treatment and at each plot, three measurements were averaged. The unidirectional‐flow flume generated a current velocity that forced the water through a 1.2 m × 0.25 m × 0.3 m (*L* × *W* × *H*) Perspex tunnel that was placed over the vegetation. The flow velocity was measured with an acoustic Doppler flow sensor (ADV, Nortek AS™ Vectrino Field Probe) that was suspended at 25 cm above the sediment surface within the flume tunnel. Two divers closely observed the sediment surface within the flume tunnel, and the critical erosion threshold was the velocity at which sediment grains situated beneath the ADV began to lift and move along the bed surface. The training was conducted before the measurements to ensure the observations were standardized. Sediment transport is proportional to flow velocity to the power of 3, therefore small changes in velocity lead to large changes in observed sediment dynamics. Visual observation of sediment movement is therefore sufficiently accurate to determine erosion thresholds for ecological studies. See James et al. (James et al., 2020) for an extensive description of the portable unidirectional‐flow flume and experimental setup. To test if grain size differences influence sediment stability among treatments and to test the relative importance of the canopy on the sediment stabilization function, we also analyzed median grain size for all plots using a Malvern Laser Particle Sizer (Figure S3). For this, we used a sub‐sample from the sediment cores (taken at 0–5 cm depth) collected for carbon storage assessment.

#### 2.4.6 Estimation of resilience to invasive species invasion

We estimated the relative rate of colonization in each experimental plot by the invasive seagrass *Halophila stipulacea*. *H. stipulacea* cover was monitored at the start and end of the experimental period in a 25 × 25 cm frame in the center of each plot. The change in cover between the start and end of the experimental period was taken as a measure of resilience to invasion for the native *T. testidinum* meadows.

### 2.5 Data analysis

All data analyses were performed in R, version 3.3.3 (R Core Team, 2017). The average aboveground seagrass (*T. testudinum*) biomass estimated at the end of the experiment was compared among the three different in situ treatments (Figure S1, Dataset S1; Christianen et al., 2022). The measured response of each ecosystem function was plotted against the aboveground biomass as the explanatory variable to represent the effects of changing megaherbivore grazing intensity. Earlier work has shown that the relationships between the structure and ecosystem functions in coastal habitats can be linear as well as nonlinear being characterized by thresholds and limiting functions (Koch et al., 2009), which in turn is relevant for the nature (thresholds, rate, level) of the response to changing megaherbivore abundance. Accordingly, we assessed the relationship between aboveground seagrass biomass and each response variable individually in five models using the *invFSxfunc* package (Angelini et al., 2015; Ramus et al., 2017). Using nonlinear least squares (Grothendieck, 2013), we fitted null, linear, log, hyperbolic, and power relationships for each response using the aboveground seagrass biomass of each plot as the explanatory variable. The selection of the best fitting model was based on the Akaike information criterion, correcting for small sample sizes (AICc) (Byrnes et al., 2014; Grothendieck, 2013). For each response variable, we compared the null model with the most probable model using a one‐way ANOVA. We reported each treatment, or aboveground seagrass biomass, as the probability (*p*) of each model, given that the null hypothesis was true. The model fit, AICc values, AICc weight, and parameter estimates for each individual ecosystem function and the multifunctionality response variable are tabulated in Dataset S2 (Christianen et al., 2022).

To assess if megaherbivore grazing intensity, reflected in treatments on aboveground seagrass biomass, had effects on the seven measured ecosystem functions, we employed the *multifunc* package (version [0.7.0]; https://github.com/jebyrnes/multifunc) as well as the averaging and single threshold approaches to quantify ecosystem multifunctionality (Byrnes et al., 2014). The averaging approach determined the average level of multiple functions by standardizing each function average to a common scale and taking the mean. Realizing that invasive seagrass cover represented a negative contribution to ecosystem multifunctionality the invasive seagrass cover was used as an inverse function. We integrated the overall effect of single ecosystem functions by estimating an average ecosystem multifunctionality index (in percent) for each plot. We assumed that high values for each of the seven functions corresponded to a high level of ecosystem function (i.e., higher values of sediment stability implied a higher performance for this function). The average ecosystem multifunctionality index can be interpreted as the average level of all seven functions. However, this index should not be used to assess whether all functions were being performed simultaneously at a high level, given that functions performed at low levels could be averaged out by those performed at high levels. Thus, we summed up the number of ecosystem functions in each plot for which the standardized estimate was above each of nine thresholds (from 10% to 90% of maximum functioning, in increments at 10%) (Byrnes et al., 2014). Threshold index scores (ranging from 0–7) denoted the number of ecosystem functions above a specific threshold in each plot.

## 3 RESULTS

The three different megaherbivore grazing intensity scenarios that were simulated by our in situ experimental treatments resulted in pronounced differences in aboveground seagrass biomass (Figure S1). Treatment 2 (intermediate turtle grazing intensity) led to reduced aboveground seagrass biomass by 55% compared to treatment 1 (no turtle grazing). Treatment 3 (intensive turtle grazing) reduced the aboveground seagrass biomass by 96%.

**FIGURE 2:**
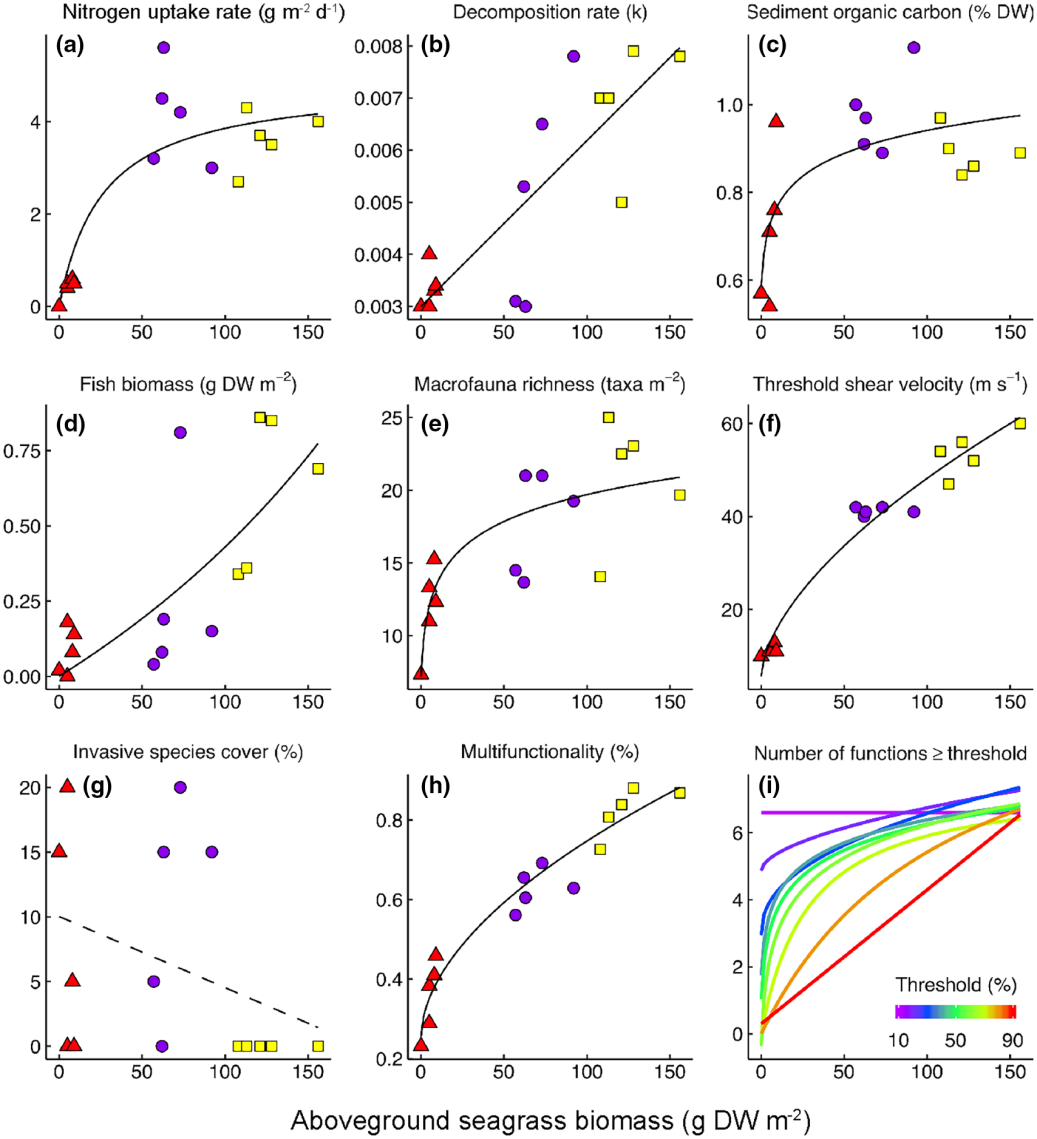
Results of experimental manipulation simulating differential megaherbivore grazing intensities on seagrass ecosystem functions and ecosystem multifunctionality, following the three megaherbivore grazing scenarios (Figure 3) with aboveground seagrass biomass as a proxy for the outcome of grazing intensity (*x*‐axis). The best fitting models determined by AICc are shown (Dataset S2; Christianen et al., 2022). (a) Net leaf nitrogen uptake rate, (b) Tea bag decomposition rate. (c) Sediment organic carbon storage. (d) Biomass of fish species. (e) The taxonomic richness of macroinvertebrates. (f) Sediment stabilization, measured as threshold shear velocity, the speed at which sediment became mobile in a unidirectional‐flow field flume. (g) Resilience against invasive species expansion, measured as change in % cover of the invasive seagrass *Halophila stipulacea (not significant)*. (h) Ecosystem multifunctionality index, the average of the seven standardized functions in percent. (i) Several functions (max seven functions) exceed threshold levels in each plot against aboveground seagrass biomass, for thresholds ranging from 10% to 90% of the maximum indicated on the color scale below. Colors and symbols correspond to the three grazing intensities Treatment 1—no turtle grazing (megaherbivores ecologically extinct, yellow squares), Treatment 2—intermediate turtle grazing (return of megaherbivores to intermediate levels, purple circles), Treatment 3—intensive turtle grazing (megaherbivores accumulation, red triangles). Solid line: significant results. Dotted line: results not significant.

The relationships between six of the seven ecosystem functions and the aboveground plant biomass (as a proxy for megaherbivore grazing intensity) were highly significant (*p* < .002, Figure 2). Nitrogen uptake, decomposition, sediment organic carbon content, fish biomass, macroinvertebrate species richness, and sediment stability were all positively related to seagrass biomass (Figure 2). Whereas the percentage invasive species cover seemed negatively related to seagrass biomass, this effect was statistically nonsignificant. We identified both linear and nonlinear relationships between aboveground seagrass biomass and individual ecosystem functions (Figure 2) and found both thresholds and saturations in the provisioning of ecosystem functions under the manipulation of seagrass biomass and grazing intensity. The response of seagrass net nitrogen uptake, underpinning the ecosystem service water purification (Table 1) was hyperbolic (Figure 2a). Decomposition and sediment organic carbon content, both functions affecting carbon storage, increased linearly (Figure 2b) and logarithmically (Figure 2c), respectively, with aboveground seagrass biomass. The response of fish biomass, a function representing the ecosystem service food provisioning, was hyperbolic (Figure 2d). The response of macroinvertebrate species richness, also representing food provisioning, to aboveground seagrass biomass, was logarithmic (Figure 2e). The response of sediment stability, a process that represents erosion control and hence coastal protection (Christianen et al., 2013), was exponential (Figure 2f), and unaffected by median grain size (Figure S3). No significant relationship between aboveground seagrass biomass and invasive species percentage was detected (Figure 2g). Markedly different effects of our treatments on ecosystem functions were also reflected in the relative responses, for example, the rate of increase in fish biomass was lower compared to the data on macroinvertebrate species richness.

**FIGURE 3:**
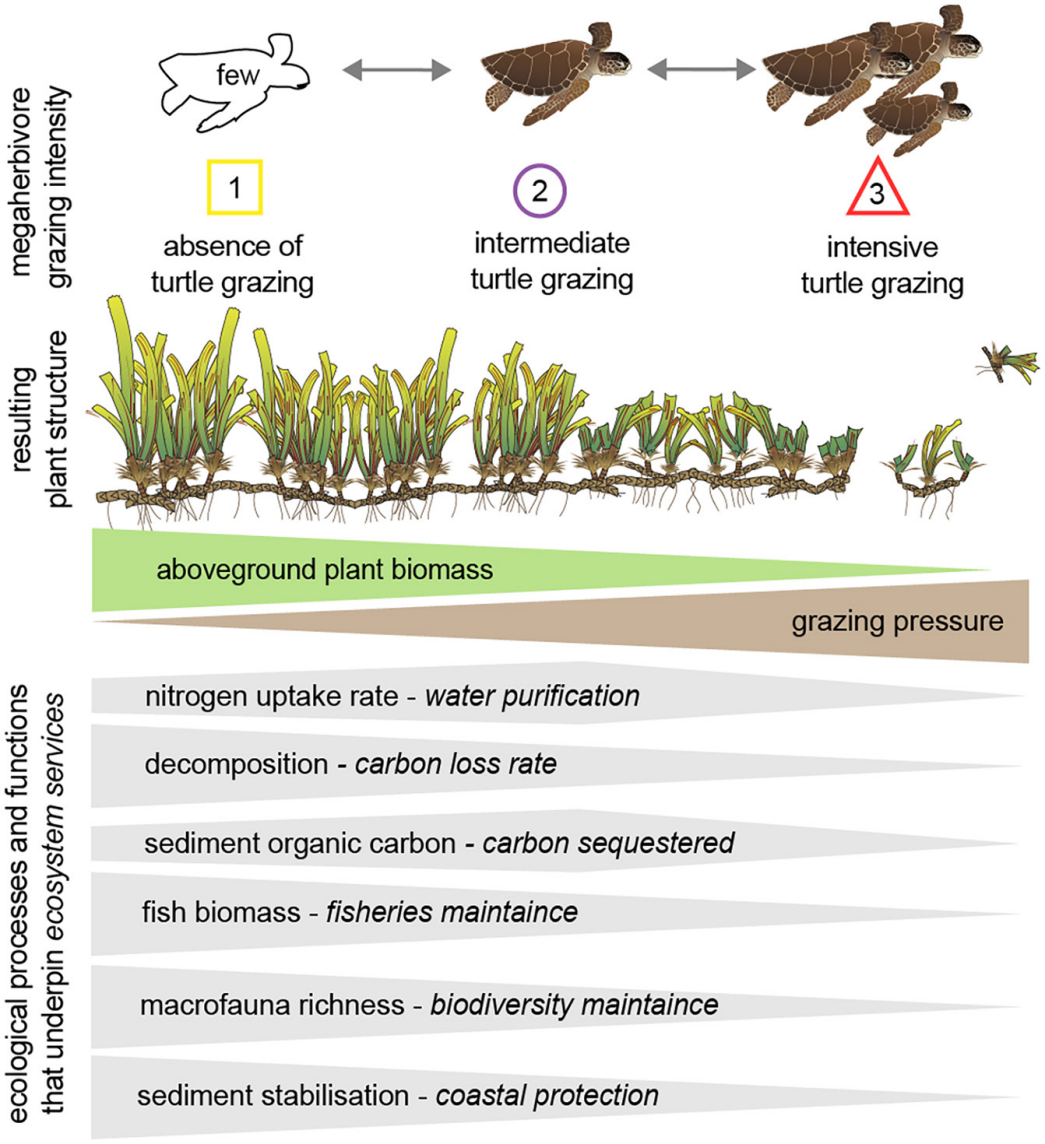
Consequences of changing marine megaherbivore densities for ecosystem functioning and services. Three scenarios of megaherbivore grazing intensity, can be observed in tropical seagrass ecosystems with green turtles as megaherbivores across the world (Figure 1b). In a *situ* experiment, three different levels of sea turtle grazing intensity were simulated as found in the literature. Treatment 1—no turtle grazing (megaherbivores ecologically extinct, yellow squares), Treatment 2—intermediate turtle grazing (return of megaherbivores to intermediate levels, purple circles), Treatment 3—intensive turtle grazing (megaherbivores accumulation, red triangles). Megaherbivore grazing intensity affects the seagrass biomass, shoot density, and canopy structure which has implications for ecosystem functioning. The impact of megaherbivore grazing intensity for single ecosystem functions and their integrated overall effect, ecosystem multifunctionality, was determined over the range of remaining seagrass biomass at the end of the 18‐month experimental period and is summarized in gray bars. Image credit (vector graphics): Joanna Woerner, Tracey Saxby, Integration and Application Network, University of Maryland Center for Environmental Science (ian.umces.edu/imagelibrary/). Images were customized by the authors.

Importantly, the impact of the simulated progressing megaherbivore intensity treatments varied among single ecosystem functions (Figure 2a–g). Under intermediate turtle grazing (Treatment 2), the plant production level was the highest and two of the seven ecosystem functions (sediment organic carbon content, and nutrient cycling) had the highest measured values. Intense grazing (Treatment 3, representing megaherbivore accumulation) resulted in a collapse of ecosystem functions. Without turtle grazing (Treatment 1), four of the seven ecosystem functions (sediment stability, fish biomass, macroinvertebrate species richness, and decomposition rate) had the highest values (Figure 2a–g).

Ecosystem multifunctionality, the combined effect of all seven single ecosystem functions, was positively related to aboveground plant biomass (Figure 2h). The effect of aboveground seagrass biomass on ecosystem multifunctionality thresholds was positive (> the 10% threshold), although the nature of the response differed among threshold values (Figure 2i); a linear relationship was observed at a 20% threshold, whereas the response was exponential at 30%, logarithmic at 40%, hyperbolic at 70%, and linear function at 90% threshold (Figure 2a–i).

## 4 DISCUSSION

In recent decades, humans have driven megafauna loss in the oceans, but have also helped some populations to rebound through successful conservation and restoration efforts (Lotze et al., 2011; Mazaris et al., 2017; McCauley et al., 2015). So far, the consequences of such changes in marine megaherbivores on ecosystem multifunctionality were poorly understood. The present study provides a novel contribution to evaluating the ecosystem multifunctionality index over a gradient of grazing intensity. We provided the first experimental evidence of strong, contrasting impacts between three different scenarios of grazing intensity on ecosystem multifunctionality by a key marine megaherbivore, the green turtle. The experimental results in one area may not necessarily apply to all seagrass systems. However, our results clearly demonstrated that while intermediate turtle grazing results in shifts in ecosystem functioning compared to turtle absence, intensive turtle grazing pressure has a disproportionally large effect on ecosystem functions and likely will result in overgrazing‐induced loss of multifunctionality. In addition, our study provides a basis for projecting historical levels of seagrass multifunctionality before their principal megaherbivores first became ecologically extinct.

### 4.1 Multifunctionality is not necessarily highest for the “natural state” of ecosystems

There have been different turtle scenarios in history (high abundance, low abundance, now sometimes hyper‐abundant), but now all three scenarios occur simultaneously in all three ocean basins where turtles are found (Figure 1b), making the outcome of this experiment globally relevant and urgent. As turtles continue to recover, this has led to the need to assess the impact of current megaherbivore scenarios on ecosystem multifunctionality (Scott et al., 2018), and to predict ecosystem impacts of future shifts. In our experiment, each separate turtle grazing intensity treatment affected ecosystem functions differently in terms of the nature and level of impact. The absence of turtle grazing increased sediment stabilization, decomposition, macrofauna species richness as well as fish biomass, but did not significantly enhance nutrient cycling and carbon storage compared to the natural grazed scenario. The largest impact was observed in the treatment corresponding to intensive grazing, leading to a loss of seagrass biomass and resulting in a simultaneous collapse of all seven ecosystem functions measured in our experimental treatments. This could eventually denude land‐ or seascape of vegetation.

What are appropriate megaherbivore densities and how close today's turtle populations are to pristine numbers is under considerable debate (Broderick et al., 2006; Christianen et al., 2021; Fløjgaard et al., 2022), but our study provides some basis for projecting historical levels of multifunctionality. Pristine seagrass meadows in the past were likely subjected to a high turtle grazing intensity until overharvesting of megafauna began with the arrival of Europeans in the Caribbean in the 17th Century with population estimations “exceeding the highest recorded wildebeest abundances in the Sergenti” (Jackson, 1997), and thus consequently lower standing biomass and higher productivity. The results presented here suggest that ecosystem multifunctionality was likely lower for pristine, grazed meadows in pre‐European times compared to contemporary seagrass meadows that are often less intensively grazed (e.g., those in Scenario 1 and 2, Figure 2). However, it should be taken into account that the ecosystem services evaluated (and the loss or gain under different treatments) are based on studies that measures in ecosystems with low presence of megafauna (both sharks and sea turtles) and contradicting effects are found. Examples of contradicting effects include some studies that have documented loss of ecosystem services due to increased grazing by green turtles in seagrass ecosystems (James et al., 2020) while others have found no effect or improvement to ecosystem services in grazed systems, including nutrient cycling, macroalgal diversity, sediment stabilization and erosion, and carbon sequestration (Christianen et al., 2012; Johnson et al., 2017, 2019, 2020; Molina Hernández & van Tussenbroek, 2014).

### 4.2 Drivers of megafauna accumulation and degradation of multifunctionality

Although many ecosystems remain depleted of megafauna (Dirzo et al., 2014), reports of rising megaherbivore populations demonstrate nature's impressive potential for resilience and the potential to reverse these declining trends (Lotze et al., 2011; McCauley et al., 2015). Green turtles are an example. Measures to protect green turtles are resulting in the rise of some populations (Chaloupka et al., 2008; Mazaris et al., 2017). However, these populations may not always find sufficiently productive habitat as local anthropogenic stress is degrading coastal habitat, including seagrass, at accelerating rates worldwide (Dunic et al., 2021; Waycott et al., 2009). In addition, tropicalization, the poleward migration of tropical herbivores due to warming water (Vergés et al., 2014), may enhance megaherbivore densities to increase or accumulate in remaining habitat and to degradation of multifunctionality. Tropicalization has brought green turtles to subtropical seagrass meadows where they were previously rare or only present in summer (Hyndes et al., 2016; Rodriguez & Heck, 2020), and where light conditions result in lower seagrass recovery rates, leading to a risk of overgrazing. Seagrass is also experiencing lower seagrass recovery rates in both tropical as in subtropical areas due to significant anthropogenic impacts to the health and stability of seagrass ecosystems, that could thereby further exacerbate the negative effects of grazing. As a result, reports of megaherbivore accumulation are becoming more frequent in areas where habitat resilience is eroding (Ballorain et al., 2010; Christianen et al., 2014; Fourqurean et al., 2019; Gangal et al., 2021; Lal et al., 2010; Molina Hernández & van Tussenbroek, 2014). In these areas megaherbivore recovery not simply alters what humans are accustomed to gaining from an unnatural, ungrazed system, but may even lead to overgrazing and the collapse of multifunctionality. An example has recently emerged in the Lakshadweep Islands where turtle overgrazing caused archipelago‐wide functional declines of seagrass meadows, with seagrass recovery being absent or low (primarily by a small pioneer species, Gangal et al., 2021).

The impact that turtle grazing has on their environment is likely accelerated further by the decline in large sharks that continues globally (Ferretti et al., 2010; Queiroz et al., 2019). Reports of seagrass overgrazing by turtles from Bermuda (Fourqurean et al., 2019) and Indonesia (Christianen et al., 2014), both show seagrass meadows where predators are ecologically extinct (Heithaus et al., 2014). Although the top‐down regulation of turtles remains a topic of debate, large sharks impact the distribution of turtles and dugongs and can reduce grazing pressure through fear effects (Burkholder et al., 2013; Heithaus, Wirsing, et al., 2008; Smulders et al., 2022; Wirsing et al., 2007), and can therefore help prevent herbivore accumulation and improve ecosystem multifunctionality. This mirrors the impact of predators on large herbivores in terrestrial system, such as wolves helping to disperse ungulates in space (Laundré et al., 2001), and in dugong grazed seagrass meadows without tiger sharks, where experiments have shown that grazing can exacerbate effects of extreme climate events on seagrass recovery and community composition (Nowicki et al., 2021). Our results imply that the enhancement of ecosystem multifunctionality requires that all ecosystem components, habitat *and*, top predators and megaherbivores recover in the same direction.

In other aquatic ecosystems ecosystem multifunctionality may be affected by similar interactions with rising megaherbivore populations, underscoring the general applicability of our findings. Examples of megaherbivore impacts include various groups of grazers. Overgrazing by Greylag goose threatened restoration of reed belts (Bakker et al., 2018), and overgrazing by waterfowl may endanger the existence of temperate seagrass meadows (Kollars et al., 2017). Overgrazing by West Indian Manatees was also shown to hinder efforts to restore submerged macrophyte beds (Hauxwell et al., 2004).

### 4.3 Integrating nonlinearity of ecosystem responses

We observed linear as well as nonlinear responses among different ecosystem services; differing in slope and saturation point, as observed previously in other coastal ecosystems (Angelini et al., 2015; Barbier et al., 2008; Koch et al., 2009; Ramus et al., 2017). We therefore echo the importance of appreciating the nonlinear response previously mentioned (Koch et al., 2009). The nonlinear responses justified our approach to measure ecosystem functions along a gradient of realized plant biomass, without which we would have failed to detect optimal responses and thresholds for the sudden collapse. Consequently, realized plant density (or biomass) proved the key parameter in assessing the impact of megaherbivore grazing intensity on ecosystem functions and services. This may likely apply to other exclosure studies as well, which to date have rarely taken into account gradients in grazing pressure or realized plant density.

### 4.4 Potential of the ecosystem multifunctionality index

Our study showcased the large potential in employing the ecosystem multifunctionality index to characterize the current and future performance of the entire ecosystem, by providing a quantitative measure of how change simultaneously influences multiple functions and services. The multifunctionality approach has been developed to investigate the relationship between ecosystem multifunctionality and biodiversity (Byrnes et al., 2014). Multifunctionality has been investigated using different methodologies for example to analyze the impact of simultaneous environmental stressors through impacts on the diversity and biomass of the community (Antiqueira et al., 2018). Here, our results on the relation between ecosystem multifunctionality and seagrass biomass changes driven by megaherbivore grazing intensity, show the potential for wider application of this approach by employing an index of multifunctionality to characterize ecosystem performance beyond biodiversity studies. However, the approach can benefit from additional developments and refinements. We revealed that the response curves differ strongly among specific ecosystem functions. In contrast to the high variability among functions underlying different ecosystem services, a low variability was found between functions underlying the same ecosystem service in our study ecosystem (Figure S2; taxonomic richness of epifauna, infauna, and fish that underly fisheries maintenance). Such low variability between functions was also found in coastal ecosystem dominated by algae (Ramus et al., 2017). Thus, the ecosystem multifunctionality index appears to be robust in terms of the choice of the specific ecosystem functions from which the index is composed. An ecosystem multifunctionality index could be applied widely across a range of habitats and ecosystems. Opportunities for further development and expansion of the ecosystem multifunctionality index include integrating a weight of each “sub‐index” or “ecosystem function” to the final ecosystem multifunctionality index and adding additional “sub‐indexes” including sociocultural and economical aspects (e.g., tourism). Tailoring the ecosystem multifunctionality index to each unique case, both by the choice of sub‐indexes as well as the weight of each sub‐index, would facilitate the application of a universal, transparent index of ecosystem performance.

### 4.5 Implications for management and conservation

Collectively, our in‐situ experiments revealed strong, contrasting impacts between three different levels of megaherbivore grazing intensity on ecosystem services and multifunctionality in a seagrass meadow, ultimately affecting human wellbeing. Our results have implications for coastal management and conservation. Building on examples of historical megafaunal declines and trophic downgrading (Estes et al., 2016), our findings make it clear that when integrative conservation approaches, aimed at top predators, megafauna and their habitats, prevent megaherbivore accumulation this may enhance ecosystem multifunctionality and restore the ecosystem functions provided by megafauna and their habitats. Unlike terrestrial systems, where there are many more protected areas but where management is now often retrospectively focused on restoring damaged habitats, in marine systems there is still a unique opportunity to proactively prevent habitat loss and reduce marine hunting to manage our impacts on marine habitats and fauna (McCauley et al., 2015). In addition, management plans need to be feasible within the constraints of the current state of affairs, rather than applying the pre‐European state as a reference (in which meadows could sustain higher numbers of megaherbivores), since this reference is no longer valid due to global decimation of megafauna, and habitat loss (Fløjgaard et al., 2022). Ecosystem interactions and dynamics must be accounted for during both the planning and management of protected areas, focusing beyond the alleviation of pressure on single species (e.g., focusing on whole seagrass ecosystems instead of green turtle conservation, Christianen et al., 2021). To arrive at balanced approaches and updated ecosystem reference states, we need a comprehensive examination of the status of the large predators, the megaherbivores and its habitats in experimental rewilding sites that need to be established. In addition, the development of new conservation and strategies also requires including nonlinear responses, habitat connectivity and dynamics, synergetic stressors, and ecosystem multifunctionality. Incorporation of integrative multifunctionality indexes toward a balanced approach to conservation and restoration has the potential to enhance ecosystem multifunctionality.

## CONFLICT OF INTEREST

The authors do not have a conflict of interest to declare.

## Supporting information


**Appendix S1:** Supporting Information

## Data Availability

The data that support the findings of this study are available on the data repository 4TU.Researchdata (https://data.4tu.nl), with the identifier https://doi.org/10.4121/21214229. Additionally, the data is included in the supporting information.
