# Supplementary material for: Seagrass ecosystem multifunctionality under the rise of a flagship marine megaherbivore
Source: Glob Chang Biol. 2022 Nov 4;29(1):215–30. doi: 10.1111/gcb.16464 (PMC10099877; doi:10.1111/gcb.16464)
Supplement: Supplementary file 1 — Appendix S1: Supporting Information [file GCB-29-215-s001.pdf]

Supporting Information for

SEAGRASS ECOSYSTEM MULTIFUNCTIONALITY UNDER THE RISE OF A FLAGSHIP MARINE MEGAHERBIVORE

Marjolijn J. A. Christianen*, Fee O. H. Smulders, Jan Arie Vonk, Leontine E. Becking, Tjeerd J. Bouma, Sabine M. Engel, Rebecca James, Mabel I. Nava, Jaco C. de Smit, Jurjan P. van der Zee, Per J. Palsbøll, Elisabeth S. Bakker

Marjolijn J. A. Christianen

*Email: marjolijn.christianen@wur.nl

The data that support the findings of this study are available on the data repository 4TU.Researchdata (https://data.4tu.nl), with the identifier https://doi.org/10.4121/21214229. Additionally, the data is included in the supporting information.

This PDF file includes:

Figs. S1 to S3

Datasets S1 to S2

Supplementary texts S1 and S2

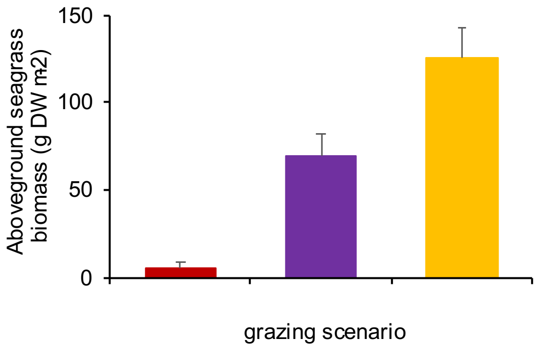

Figure S1. **The average aboveground seagrass biomass (± SE) measured at the end of the experiment** showed significant differences among the three megaherbivore grazing scenarios (ANOVA; $F_{2,12}$ = 94.6, P < 0.0001, R^2 = 0.94). Yellow - no turtle grazing (megaherbivores ecologically extinct), Purple - intermediate turtle grazing (return of megaherbivores to intermediate levels), Red - intensive turtle grazing.

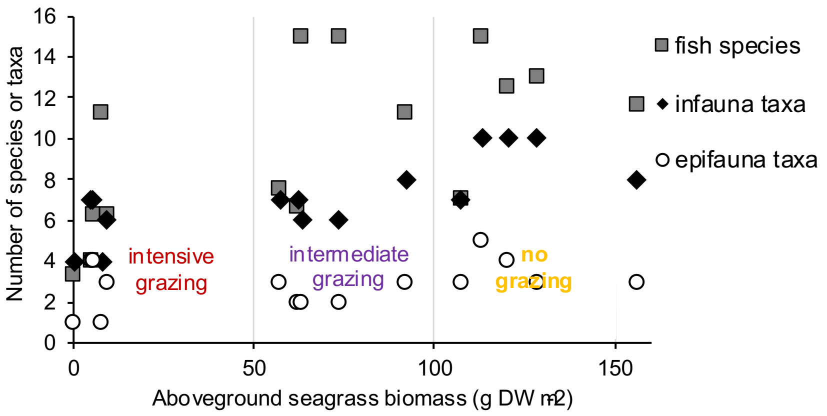

Figure S2. The taxonomic richness of fish, infauna, and epifauna taxa along the gradient in seagrass aboveground biomass of the treatments.

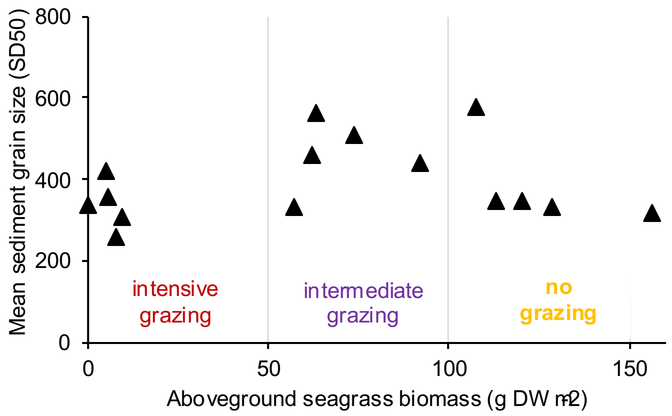

Figure S3. Sediment median grain size was comparable along the seagrass biomass gradient in the treatments ($P > 0.05$, $R^2 = 0.02$). Therefore, sediment grain size appeared uncorrelated with the sediment stabilization proxies ($P > 0.05$) and could not explain the differences in threshold shear velocities (Fig. 2f).

Dataset S1. Mean plot-level responses used in all analyses. Column names: **Plot,** number of the experimental plot; **d_scen**, megaherbivore grazing intensity levels, numbers correspond with numbers in Fig. 2; **TtA**, the aboveground biomass of *Thalassia testudinum* at the end of the experiment (g DW m^{-2}); **TtL.prN**, the net leaf nitrogen uptake rate of *T. testudinum* at the end of the experiment (g N m^{-2} d^{-1}); **Krate.dec**, decomposition rate (*k*); **sed.OC**, sediment organic carbon content (%); **fish.Spec**, number of fish species (# m^{-2}); **fish.biom**, fish biomass (g DW m^{-2}); **ep.in.Tax**, richness of macroinvertebrate taxa, the sum of infauna and epifauna taxa (# m^{-2}). **u.SedMob**, speed at which sediment was mobile in a unilateral field flume (m s^{-1}); **SD50**, median sediment grain size (µm SD50); **Hs.cov**, area cover of invasive *Halophila stipulacea* (%).

plot	d_scen	TtA	TtL.prN	Krate.dec	sed.OC	fish.Spec	fish.biom	ep.in.Tax	u.SedMob	SD50	Hs.cov
26	2	0	0.0	0.003	0.57	3.33	0.02	4	10	338	15
29	2	5	0.4	0.004	0.54	4	0.00	7	11	421	0
27	2	5	0.5	0.003	0.71	6.32	0.18	7	11	359	20
28	2	8	0.6	0.0033	0.76	11.25	0.08	4	13	262	5
30	2	9	0.5	0.0034	0.96	6.32	0.14	6	11	307	0
12	1	57	3.2	0.0031	1	7.5	0.04	7	42	332	5
13	1	62	4.5	0.0053	0.91	6.67	0.08	7	40	460	0
15	1	63	5.6	0.003	0.97	15	0.19	6	41	563	15
14	1	73	4.2	0.0065	0.89	15	0.81	6	42	507	20
11	1	92	3.0	0.0078	1.13	11.25	0.15	8	41	439	15
22	3	108	2.7	0.007	0.97	7.06	0.34	7	54	577	0
24	3	113	4.3	0.007	0.9	15	0.36	10	47	346	0
23	3	121	3.7	0.005	0.84	12.5	0.86	10	56	350	0
25	3	128	3.5	0.0079	0.86	13.04	0.85	10	52	332	0
21	3	156	4.0	0.0078	0.89	11.665	0.69	8	60	321	0

Dataset S2. Model selection tables for all response variables and all models considered. Column names: **Response variable**, the dependent variable; **Model**, name describing the type of model fitted per response variable; **Formula**, the model formula used (in R notation), where 'TtA' refers to *Thalassia testudinum* aboveground biomass, the independent variable; **K**, number of estimated parameters in the model; **a**, parameter estimate of coefficient a in the model formula; **b**, parameter estimate of coefficient b in the model formula; **c**, parameter estimate of coefficient c in the model formula; **AICc**, Akaike information criterion, corrected for small sample sizes; **ΔAICc**, the difference between AICc and the lowest AICc value in the set of models considered for a given response variable; **AICcWt**, the AICc weights; **LL**, maximized log-likelihood of the model, given the data; **df**, degrees of freedom in the fitted model; **F**, F-test statistic resulting from one-way ANOVA comparing the estimated model to the null model for a given response variable; **P**, the probability of obtaining the estimated model, given that the null model is true; **adjR2**, proportion of variance in the response variable explained by *T. testudinum* aboveground biomass in estimated model adjusted for the number of parameters used in the model (K).

Response.variable	Model	Formula	K	a	b	c	AICc	ΔAICc	AICcWt	LL	df	F	P	adjR2
Net nitrogen uptake rate (g m-2 d-1)	Hyperbolic	a * TtA/(b + TtA)	3	4.9	26.0	NA	44.6	0.0	0.812	-18.2	13	48.2	<0.001	0.77
Net nitrogen uptake rate (g m-2 d-1)	Log	a + b * log(TtA + 1)	3	-0.9	1.0	NA	47.7	3.1	0.172	-19.8	13	36.8	<0.001	0.72
Net nitrogen uptake rate (g m-2 d-1)	Power	a + b * TtA^c	4	-0.5	0.8	0.4	53.0	8.5	0.012	-20.5	12	14.7	0.001	0.66
Net nitrogen uptake rate (g m-2 d-1)	Linear	a + b * TtA	3	1.0	0.0	NA	55.7	11.1	0.003	-23.8	13	16.1	0.001	0.52
Net nitrogen uptake rate (g m-2 d-1)	Null	a	2	2.7	NA	NA	57.6	13.1	0.001	-29.8	14	0.0	1.000	0.00
Decomposition rate (k)	Linear	a + b * TtA	3	0.0	0.0	NA	-154.4	0.0	0.820	81.3	13	29.3	<0.001	0.67
Decomposition rate (k)	Power	a + b * TtA^c	4	0.0	0.0	1.2	-150.7	3.7	0.131	81.4	12	13.7	0.001	0.65
Decomposition rate (k)	Log	a + b * log(TtA + 1)	3	0.0	0.0	NA	-147.8	6.6	0.030	78.0	13	14.3	0.002	0.49
Decomposition rate (k)	Null	a	2	0.0	NA	NA	-146.9	7.5	0.019	72.4	14	0.0	1.000	0.00
Decomposition rate (k)	Hyperbolic	a * TtA/(b + TtA)	3	0.0	-29.4	NA	-120.7	33.7	0.000	64.4	13	-8.5	1.000	-2.13
Sediment organic carbon (%)	Log	a + b * log(TtA + 1)	3	0.6	0.1	NA	-17.5	0.0	0.648	12.8	13	15.8	0.002	0.51
Sediment organic carbon (%)	Null	a	2	0.9	NA	NA	-15.7	1.7	0.271	6.9	14	0.0	1.000	0.00
Sediment organic carbon (%)	Power	a + b * TtA^c	4	0.6	0.1	0.2	-12.7	4.7	0.061	12.4	12	6.5	0.012	0.44
Sediment organic carbon (%)	Linear	a + b * TtA	3	0.8	0.0	NA	-10.5	7.0	0.020	9.3	13	5.1	0.042	0.23
Sediment organic carbon (%)	Hyperbolic	a * TtA/(b + TtA)	3	1.0	2.0	NA	-2.0	15.4	0.000	5.1	13	-2.7	1.000	-0.36
Fisheries biomass (g DW m-2)	Hyperbolic	a * TtA/(b + TtA)	3	-1.8	-508.3	NA	3.3	0.0	0.405	2.5	13	16.5	0.001	0.53
Fisheries biomass (g DW m-2)	Linear	a + b * TtA	3	0.0	0.0	NA	3.6	0.3	0.344	2.3	13	15.9	0.002	0.52
Fisheries biomass (g DW m-2)	Null	a	2	0.3	NA	NA	5.4	2.1	0.142	-3.7	14	0.0	1.000	0.00
Fisheries biomass (g DW m-2)	Power	a + b * TtA^c	4	0.1	0.0	1.6	6.6	3.3	0.076	2.7	12	8.0	0.006	0.50
Fisheries biomass (g DW m-2)	Log	a + b * log(TtA + 1)	3	-0.1	0.1	NA	8.3	5.0	0.033	-0.1	13	8.1	0.014	0.34
Macroinvertebrate richness (taxa m-2)	Log	a + b * log(TtA + 1)	3	7.2	2.7	NA	83.0	0.0	0.633	-37.4	13	24.3	<0.001	0.63
Macroinvertebrate richness (taxa m-2)	Linear	a + b * TtA	3	11.8	0.1	NA	85.5	2.5	0.183	-38.6	13	18.6	0.001	0.56
Macroinvertebrate richness (taxa m-2)	Power	a + b * TtA^c	4	7.6	2.8	0.3	86.3	3.3	0.122	-37.1	12	11.8	0.001	0.61
Macroinvertebrate richness (taxa m-2)	Null	a	2	16.9	NA	NA	88.6	5.6	0.038	-45.3	14	0.0	1.000	0.00
Macroinvertebrate richness (taxa m-2)	Hyperbolic	a * TtA/(b + TtA)	3	20.3	3.8	NA	89.5	6.5	0.024	-40.7	13	11.1	0.005	0.42
Threshold flow velocity (m s-1)	Power	a + b * TtA^c	4	5.7	2.7	0.6	91.9	0.0	0.688	-40.0	12	156.3	<0.001	0.96
Threshold flow velocity (m s-1)	Hyperbolic	a * TtA/(b + TtA)	3	71.1	44.3	NA	93.7	1.7	0.287	-42.7	13	229.6	<0.001	0.94
Threshold flow velocity (m s-1)	Linear	a + b * TtA	3	12.4	0.3	NA	98.6	6.7	0.024	-45.2	13	161.2	<0.001	0.92
Threshold flow velocity (m s-1)	Log	a + b * log(TtA + 1)	3	-5.3	11.4	NA	105.9	14.0	0.001	-48.9	13	94.3	<0.001	0.87
Threshold flow velocity (m s-1)	Null	a	2	35.4	NA	NA	127.4	35.5	0.000	-64.7	14	0.0	1.000	0.00
Invasive species cover (%)	Null	a	2	6.3	NA	NA	102.4	0.0	0.965	-52.2	14	0.0	1.000	0.00
Invasive species cover (%)	Linear	a + b * TtA	3	10.0	-0.1	NA	110.5	8.2	0.016	-51.2	13	1.9	0.194	0.06

Response.variable	Model	Formula	K	a	b	c	AICc	ΔAICc	AICcWt	LL	df	F	P	adjR2
Invasive species cover (%)	Log	a + b * log(TtA + 1)	3	12.2	-1.6	NA	111.0	8.7	0.013	-51.4	13	1.4	0.261	0.03
Invasive species cover (%)	Power	a + b * TtA^c	4	9.0	0.0	2.9	113.3	11.0	0.004	-50.7	12	1.3	0.297	0.05
Invasive species cover (%)	Hyperbolic	a * TtA/(b + TtA)	3	5.1	-1.8	NA	114.6	12.2	0.002	-53.2	13	-1.6	1.000	-0.23
Multifunctionality (%)	Power	a + b * TtA^c	4	0.3	0.0	0.5	-36.8	0.0	0.754	24.4	12	102.9	<0.001	0.94
Multifunctionality (%)	Linear	a + b * TtA	3	0.3	0.0	NA	-34.4	2.4	0.224	21.3	13	142.6	<0.001	0.91
Multifunctionality (%)	Log	a + b * log(TtA + 1)	3	0.1	0.1	NA	-29.7	7.1	0.021	18.9	13	100.8	<0.001	0.88
Multifunctionality (%)	Hyperbolic	a * TtA/(b + TtA)	3	0.8	8.0	NA	-18.6	18.2	0.000	13.4	13	41.5	<0.001	0.74
Multifunctionality (%)	Null	a	2	0.6	NA	NA	-7.3	29.5	0.000	2.7	14	0.0	1.000	0.00
Nr. functions above 10% threshold	Null	a	2	6.6	NA	NA	25.8	0.0	0.368	-13.9	14	0.0	1.000	0.00
Nr. functions above 10% threshold	Log	a + b * log(TtA + 1)	3	5.6	0.3	NA	26.1	0.3	0.312	-9.0	13	12.1	0.004	0.44
Nr. functions above 10% threshold	Power	a + b * TtA^c	4	5.0	1.2	0.1	26.3	0.5	0.281	-7.2	12	8.7	0.005	0.52
Nr. functions above 10% threshold	Linear	a + b * TtA	3	6.1	0.0	NA	30.3	4.5	0.039	-11.0	13	6.0	0.029	0.26
Nr. functions above 10% threshold	Hyperbolic	a * TtA/(b + TtA)	3	6.9	0.5	NA	59.7	33.9	0.000	-25.8	13	-10.3	1.000	-4.23
Nr. functions above 20% threshold	Linear	a + b * TtA	3	5.2	0.0	NA	21.2	0.0	0.433	-6.5	13	51.5	<0.001	0.78
Nr. functions above 20% threshold	Power	a + b * TtA^c	4	4.9	0.1	0.6	21.8	0.5	0.332	-4.9	12	31.1	<0.001	0.81
Nr. functions above 20% threshold	Log	a + b * log(TtA + 1)	3	4.4	0.5	NA	22.4	1.2	0.235	-7.1	13	46.5	<0.001	0.77
Nr. functions above 20% threshold	Null	a	2	6.2	NA	NA	35.1	13.9	0.000	-18.5	14	0.0	1.000	0.00
Nr. functions above 20% threshold	Hyperbolic	a * TtA/(b + TtA)	3	6.9	2.0	NA	59.7	38.4	0.000	-25.7	13	-8.0	1.000	-1.81
Nr. functions above 30% threshold	Power	a + b * TtA^c	4	2.9	0.5	0.4	7.4	0.0	0.987	2.3	12	250.3	<0.001	0.97
Nr. functions above 30% threshold	Log	a + b * log(TtA + 1)	3	2.4	0.9	NA	16.0	8.6	0.013	-3.9	13	229.4	<0.001	0.94
Nr. functions above 30% threshold	Linear	a + b * TtA	3	3.9	0.0	NA	25.8	18.4	0.000	-8.8	13	112.9	<0.001	0.89
Nr. functions above 30% threshold	Hyperbolic	a * TtA/(b + TtA)	3	6.9	4.8	NA	46.7	39.3	0.000	-19.2	13	18.4	0.001	0.55
Nr. functions above 30% threshold	Null	a	2	5.6	NA	NA	49.7	42.3	0.000	-25.9	14	0.0	1.000	0.00
Nr. functions above 40% threshold	Log	a + b * log(TtA + 1)	3	1.7	1.0	NA	31.5	0.0	0.670	-11.7	13	103.8	<0.001	0.88
Nr. functions above 40% threshold	Power	a + b * TtA^c	4	2.0	0.8	0.4	33.0	1.5	0.313	-10.5	12	56.8	<0.001	0.89
Nr. functions above 40% threshold	Linear	a + b * TtA	3	3.4	0.0	NA	39.1	7.6	0.015	-15.4	13	57.5	<0.001	0.80
Nr. functions above 40% threshold	Hyperbolic	a * TtA/(b + TtA)	3	6.7	5.4	NA	44.3	12.8	0.001	-18.1	13	36.7	<0.001	0.72
Nr. functions above 40% threshold	Null	a	2	5.3	NA	NA	54.2	22.8	0.000	-28.1	14	0.0	1.000	0.00
Nr. functions above 50% threshold	Log	a + b * log(TtA + 1)	3	1.0	1.1	NA	42.7	0.0	0.654	-17.3	13	62.3	<0.001	0.81
Nr. functions above 50% threshold	Power	a + b * TtA^c	4	1.1	1.2	0.3	44.8	2.1	0.230	-16.4	12	33.0	<0.001	0.82
Nr. functions above 50% threshold	Linear	a + b * TtA	3	2.9	0.0	NA	47.4	4.6	0.064	-19.6	13	42.3	<0.001	0.75
Nr. functions above 50% threshold	Hyperbolic	a * TtA/(b + TtA)	3	6.6	6.4	NA	47.8	5.1	0.052	-19.8	13	40.8	<0.001	0.74
Nr. functions above 50% threshold	Null	a	2	5.1	NA	NA	58.9	16.2	0.000	-30.5	14	0.0	1.000	0.00
Nr. functions above 60% threshold	Log	a + b * log(TtA + 1)	3	-0.4	1.4	NA	36.3	0.0	0.567	-14.1	13	152.6	<0.001	0.92
Nr. functions above 60% threshold	Power	a + b * TtA^c	4	0.0	1.2	0.4	37.3	1.0	0.347	-12.6	12	86.3	<0.001	0.92
Nr. functions above 60% threshold	Hyperbolic	a * TtA/(b + TtA)	3	7.2	14.8	NA	40.1	3.8	0.084	-16.0	13	115.3	<0.001	0.89
Nr. functions above 60% threshold	Linear	a + b * TtA	3	2.1	0.0	NA	47.3	11.0	0.002	-19.5	13	66.7	<0.001	0.82
Nr. functions above 60% threshold	Null	a	2	4.7	NA	NA	64.3	28.0	0.000	-33.1	14	0.0	1.000	0.00
Nr. functions above 70% threshold	Hyperbolic	a * TtA/(b + TtA)	3	7.6	28.7	NA	46.0	0.0	0.430	-18.9	13	83.2	<0.001	0.85
Nr. functions above 70% threshold	Log	a + b * log(TtA + 1)	3	-0.9	1.5	NA	46.6	0.6	0.325	-19.2	13	79.7	<0.001	0.85
Nr. functions above 70% threshold	Power	a + b * TtA^c	4	-0.1	0.8	0.4	47.5	1.5	0.199	-17.8	12	45.7	<0.001	0.87
Nr. functions above 70% threshold	Linear	a + b * TtA	3	1.5	0.0	NA	50.5	4.5	0.046	-21.1	13	58.5	<0.001	0.80
Nr. functions above 70% threshold	Null	a	2	4.3	NA	NA	65.9	19.8	0.000	-33.9	14	0.0	1.000	0.00
Nr. functions above 80% threshold	Hyperbolic	a * TtA/(b + TtA)	3	11.6	114.2	NA	46.2	0.0	0.721	-19.0	13	94.3	<0.001	0.87
Nr. functions above 80% threshold	Linear	a + b * TtA	3	0.7	0.0	NA	49.4	3.2	0.144	-20.6	13	73.6	<0.001	0.84
Nr. functions above 80% threshold	Power	a + b * TtA^c	4	-0.2	0.4	0.6	49.9	3.7	0.112	-19.0	12	43.8	<0.001	0.86
Nr. functions above 80% threshold	Log	a + b * log(TtA + 1)	3	-1.7	1.5	NA	53.0	6.8	0.024	-22.4	13	55.2	<0.001	0.80
Nr. functions above 80% threshold	Null	a	2	3.7	NA	NA	67.7	21.5	0.000	-34.8	14	0.0	1.000	0.00
Nr. functions above 90% threshold	Linear	a + b * TtA	3	0.3	0.0	NA	46.9	0.0	0.526	-19.3	13	70.1	<0.001	0.83
Nr. functions above 90% threshold	Hyperbolic	a * TtA/(b + TtA)	3	125.6	2784.7	NA	47.5	0.6	0.391	-19.6	13	66.9	<0.001	0.83
Nr. functions above 90% threshold	Power	a + b * TtA^c	4	0.4	0.0	1.1	50.6	3.8	0.080	-19.3	12	32.5	<0.001	0.82
Nr. functions above 90% threshold	Log	a + b * log(TtA + 1)	3	-1.5	1.2	NA	57.1	10.2	0.003	-24.5	13	29.0	<0.001	0.67
Nr. functions above 90% threshold	Null	a	2	3.0	NA	NA	64.5	17.7	0.000	-33.3	14	0.0	1.000	0.00

SI text 1

Literature survey global megaherbivore grazing scenarios

To analyse the number of publications on seagrass and megaherbivores in peer-reviewed journals over time a survey was performed on literature published from 1960 to June 2022 using the search terms "seagrass* AND turtle AND (graz* OR herb*)" using SCOPUS, and Web of Science. 148 peer-reviewed publications were identified that address seagrass and turtle grazing in some way (*SI text 2),* the first publication is from 1978. Since 1996, more than one publication per year has been published, and the publication rate rapidly increased from 4 per year in 2016 to 16 per year in 2021 (Fig. 1a).

To illustrate the distribution of the three different grazing scenarios for green turtles, an example of all three scenarios was selected from literature for each of the three ocean basins where green turtles and (sub)tropical seagrass co-occur. And these were presented in a map (Fig. 1b). The map is not complete but shows that all three grazing scenarios can currently still be found in multiple ocean basins, making the outcome of our experiment globally relevant and urgent.

SI text 2
References literature survey

Authors	Title	Year	Source title	Volume	Issue	Page s	Page e	DOI
Meylan, PA; Hardy, RF; Gray, JA; Meylan, AB	A half-century of demographic changes in a green turtle (Chelonia mydas) foraging aggregation during an era of seagrass decline	2022	Marine Biology	169	6			10.1007/s00227-022-04056-5
Bastos, KV; Machado, LP; Joyeux, J-C; Ferreira, JS; Militão, FP; Fernandes, VDO; Santos, RG	Coastal degradation impacts on green turtle's (Chelonia mydas) diet in southeastern Brazil: Nutritional richness and health	2022	Science of the Total Environment	823				10.1016/j.scitotenv.2022.153593
Kale, N; Manoharakrishnan, M; Bharti, DK; Poti, M; Shanker, K	The island hoppers: how foraging influences green turtle Chelonia mydas abundance over space and time in the Lakshadweep Archipelago, India	2022	Endangered Species Research	48		1	14	10.3354/esr01181
Wang, XY; Bai, JH; Yan, JG; Cui, BS; Shao, DD	How Turbidity Mediates the Combined Effects of Nutrient Enrichment and Herbivory on Seagrass Ecosystems	2022	Frontiers in Marine Science	9				10.3389/fmars.2022.787041
Tol, SJ; Jarvis, JC; York, PH; Congdon, BC; Coles, RG	Mutualistic relationships in marine angiosperms: Enhanced germination of seeds by mega-herbivores	2021	Biotropica	53	6	1535	1545	10.1111/btp.13001
Gulick, AG; Meylan, AB; Meylan, PA; Hart, KM; Gray, JA; Roth, G; Bolten, AB; Bjorndal, KA	Role of ingesta particle size in the green turtle grazing strategy, ontogenetic diet shifts, and responses to seagrass declines	2021	Marine Biology	168	10			10.1007/s00227-021-03965-1
Buckee, J; Hetzel, Y; Nyegaard, M; Evans, S; Whiting, S; Scott, S; Ayvazian, S; van Keulen, M; Verduin, J	Catastrophic loss of tropical seagrass habitats at the Cocos (Keeling) Islands due to multiple stressors	2021	Marine Pollution Bulletin	170				10.1016/j.marpolbul.2021.112602
Troyo, A; Jiménez-Duran, K; Van Tussenbroek, BI; Márquez-Guzmán, J; Wong, JGR; Corona-Carrillo, JI; Díaz-Pontones, DM	Fruit development in the seagrass Thalassia testudinum: Possible relationships between structure, physiology and defense	2021	Aquatic Botany	174				10.1016/j.aquabot.2021.103418
Gulick, AG; Johnson, RA; Pollock, CG; Hillis-Starr, Z; Bolten, AB; Bjorndal, KA	Recovery of a cultivation grazer: A mechanism for compensatory growth of Thalassia testudinum in a Caribbean seagrass meadow grazed by green turtles	2021	Journal of Ecology	109	8	3031	3045	10.1111/1365-2745.13718
Gangal, M; Gafoor, A-B; D'Souza, E; Kelkar, N; Karkarey, R; Marbà, N; Arthur, R; Alcoverro, T	Sequential overgrazing by green turtles causes archipelago-wide functional extinctions of seagrass meadows	2021	Biological Conservation	260				10.1016/j.biocon.2021.109195
Long, CA; Chabot, RM; El-Khazen, MN; Kelley, JR; Mollet-Saint Benoît, C; Mansfield, KL	Incongruent long-term trends of a marine consumer and primary producers in a habitat affected by nutrient pollution	2021	Ecosphere	12	6			10.1002/ecs2.3553
Palmer, JL; Beton, D; Çiçek, BA; Davey, S; Duncan, EM; Fuller, WJ; Godley, BJ; Haywood, JC; Hüseyinoğlu, MF; Omeyer, LCM; Schneider, MJ; Snape, RTE; Broderick, AC	Dietary analysis of two sympatric marine turtle species in the eastern Mediterranean	2021	Marine Biology	168	6			10.1007/s00227-021-03895-y
Rodriguez, AR; Heck, KL	Approaching a Tipping Point? Herbivore Carrying Capacity Estimates in a Rapidly Changing, Seagrass-Dominated Florida Bay	2021	Estuaries and Coasts	44	2	522	534	10.1007/s12237-020-00866-2
Scott, AL; York, PH; Rasheed, MA	Herbivory Has a Major Influence on Structure and Condition of a Great Barrier Reef Subtropical Seagrass Meadow	2021	Estuaries and Coasts	44	2	506	521	10.1007/s12237-020-00868-0
Scott, AL; York, PH; Macreadie, PI; Rasheed, MA	Spatial and temporal variability of green turtle and dugong herbivory in seagrass meadows of the southern Great Barrier Reef	2021	Marine Ecology Progress Series	667		225	231	10.3354/meps13703
Rezaie-Atagholipour, M; Imani, F; Ghezellou, P; Seminoff, JA	Feeding ecology of juvenile green turtles in food-poor habitats of the Persian Gulf	2021	Marine Biology	168	1			10.1007/s00227-020-03809-4
Inoue, H; Mizutani, A; Nanjo, K; Tsutsumi, K; Kohno, H	Fish assemblage structure response to seagrass bed degradation due to overgrazing by the green sea turtle Chelonia mydas at Iriomote Island, southern Japan	2021	Ichthyological Research	68	1	111	125	10.1007/s10228-020-00775-1
Valentine, JF; Heck, KL	Herbivory in Seagrass Meadows: an Evolving Paradigm	2021	Estuaries and Coasts	44	2	491	505	10.1007/s12237-020-00849-3
Vanderklift, MA; Pillans, RD; Hutton, M; De Weyer, L; Kendrick, GA; Zavala-Perez, A; Verges, A; Garthwin, R; Oades, D; McCarthy, P; George, K; Sampi, T; George, D; Sampi, C; Edgar, Z; Dougal, K; Howard, A	High rates of herbivory in remote northwest Australian seagrass meadows by rabbitfish and green turtles	2021	Marine Ecology Progress Series	665		63	73	10.3354/meps13657
Lee, CL; Lin, WJ; Liu, PJ; Shao, KT; Lin, HJ	Highly Productive Tropical Seagrass Beds Support Diverse Consumers and a Large Organic Carbon Pool in the Sediments	2021	Diversity	13	11			10.3390/d13110544
Esteban, N; Mortimer, JA; Stokes, HJ; Laloë, J-O; Unsworth, RKF; Hays, GC	A global review of green turtle diet: sea surface temperature as a potential driver of omnivory levels	2020	Marine Biology	167	12			10.1007/s00227-020-03786-8
Scott, AL; York, PH; Rasheed, MA	Green turtle (Chelonia mydas) grazing plot formation creates structural changes in a multi-species Great Barrier Reef seagrass meadow	2020	Marine Environmental Research	162				10.1016/j.marenvres.2020.105183
Gulick, AG; Johnson, RA; Pollock, CG; Hillis-Starr, Z; Bolten, AB; Bjorndal, KA	Recovery of a large herbivore changes regulation of seagrass productivity in a naturally grazed Caribbean ecosystem	2020	Ecology	101	12			10.1002/ecy.3180
Leemans, L; Martínez, I; van der Heide, T; van Katwijk, MM; van Tussenbroek, BI	A Mutualism Between Unattached Coralline Algae and Seagrasses Prevents Overgrazing by Sea Turtles	2020	Ecosystems	23	8	1631	1642	10.1007/s10021-020-00492-w
Saragoça Bruno, R; Restrepo, JA; Valverde, RA	Effects of El Niño Southern Oscillation and local ocean temperature on the reproductive output of green turtles (Chelonia mydas) nesting at Tortuguero, Costa Rica	2020	Marine Biology	167	9			10.1007/s00227-020-03749-z
Barbosa, M; Taylor, CM	Spatial and Temporal Trends in Diet for Pinfish (Lagodon rhomboides) from Turtle Grass (Thalassia testudinum) Beds with Contrasting Environmental Regimes in the Lower Laguna Madre, Texas	2020	Estuaries and Coasts	43	6	1571	1581	10.1007/s12237-020-00717-0
Johnson, RA; Gulick, AG; Constant, N; Bolten, AB; Smulders, FOH; Christianen, MJA; Nava, MI; Kolasa, K; Bjorndal, KA	Seagrass ecosystem metabolic carbon capture in response to green turtle grazing across Caribbean meadows	2020	Journal of Ecology	108	3	1101	1114	10.1111/1365-2745.13306
Rodriguez, AR; Heck, KL	Green turtle herbivory and its effects on the warm, temperate seagrass meadows of St. Joseph Bay, Florida (USA)	2020	Marine Ecology Progress Series	639		37	51	10.3354/meps13285
Vanderklift, MA; Babcock, RC; Barnes, PB; Cresswell, AK; Feng, M; Haywood, MDE; Holmes, TH; Lavery, PS; Pillans, RD; Smallwood, CB; Thomson, DP; Tucker, AD; Waples, K; Wilson, SK	The oceanography and marine ecology of ningaloo, a world heritage area	2020	Oceanography and Marine Biology	58		143	178	10.1201/9780429351495-4
Cardona, L; Campos, P; Velásquez-Vacca, A	Contribution of green turtles Chelonia mydas to total herbivore biomass in shallow tropical reefs of oceanic islands	2020	PLoS ONE	15	1			10.1371/journal.pone.0228548
Bloodgood, JCG; Hernandez, SM; Isaiah, A; Suchodolski, JS; Hoopes, LA; Thompson, PM; Waltzek, TB; Norton, TM	The effect of diet on the gastrointestinal microbiome of juvenile rehabilitating green turtles (Chelonia mydas)	2020	PLoS ONE	15	1			10.1371/journal.pone.0227060
Johnson, RA; Hanes, KM; Bolten, AB; Bjorndal, KA	Simulated green turtle grazing affects benthic infauna abundance and community composition but not diversity in a Thalassia testudinum seagrass meadow	2020	Journal of Experimental Marine Biology and Ecology	522				10.1016/j.jembe.2019.151266
Johnson, RA; Gulick, AG; Bolten, AB; Bjorndal, KA	Rates of Sediment Resuspension and Erosion Following Green Turtle Grazing in a Shallow Caribbean Thalassia testudinum Meadow	2019	Ecosystems	22	8	1787	1802	10.1007/s10021-019-00372-y
Whitman, ER; Heithaus, MR; Barcia, LG; Brito, DN; Rinaldi, C; Kiszka, JJ	Effect of seagrass nutrient content and relative abundance on the foraging behavior of green turtles in the face of a marine plant invasion	2019	Marine Ecology Progress Series	628		171	182	10.3354/meps13092
Stokes, HJ; Mortimer, JA; Hays, GC; Unsworth, RKF; Laloë, J-O; Esteban, N	Green turtle diet is dominated by seagrass in the Western Indian Ocean except amongst gravid females	2019	Marine Biology	166	10			10.1007/s00227-019-3584-3
Fourqurean, JW; Manuel, SA; Coates, KA; Massey, SC; Kenworthy, WJ	Decadal Monitoring in Bermuda Shows a Widespread Loss of Seagrasses Attributable to Overgrazing by the Green Sea Turtle Chelonia mydas	2019	Estuaries and Coasts	42	6	1524	1540	10.1007/s12237-019-00587-1
López, IGM; Van Den Akker, M; Walk, L; Van Katwijk, MM; Van Der Heide, T; Van Tussenbroek, BI	Nutrient availability induces community shifts in seagrass meadows grazed by turtles	2019	PeerJ	2019	9			10.7717/peerj.7570
Hearne, EL; Johnson, RA; Gulick, AG; Candelmo, A; Bolten, AB; Bjorndal, KA	Effects of green turtle grazing on seagrass and macroalgae diversity vary spatially among seagrass meadows	2019	Aquatic Botany	152		10	15	10.1016/j.aquabot.2018.09.005
Christianen, MJA; Smulders, FOH; Engel, MS; Nava, MI; Willis, S; Debrot, AO; Palsbøll, PJ; Vonk, JA; Becking, LE	Megaherbivores may impact expansion of invasive seagrass in the Caribbean	2019	Journal of Ecology	107	1	45	57	10.1111/1365-2745.13021
Bjorndal, KA; Bolten, AB; Chaloupka, M	Green turtle somatic growth dynamics: distributional regression reveals effects of differential emigration	2019	Marine Ecology Progress Series	616		185	195	10.3354/meps12946
Goss, H; Jaskiel, J; Rotjan, R	Thalassia testudinum as a potential vector for incorporating microplastics into benthic marine food webs	2018	Marine Pollution Bulletin	135		1085	1089	10.1016/j.marpolbul.2018.08.024
Thomson, JA; Whitman, ER; Garcia-Rojas, MI; Bellgrove, A; Ekins, M; Hays, GC; Heithaus, MR	Individual specialization in a migratory grazer reflects long-term diet selectivity on a foraging ground: implications for isotope-based tracking	2018	Oecologia	188	2	429	439	10.1007/s00442-018-4218-z
Hancock, JM; Vieira, S; Jimenez, V; Rio, JC; Rebelo, R	Stable isotopes reveal dietary differences and site fidelity in juvenile green turtles foraging around São Tomé Island, West Central Africa	2018	Marine Ecology Progress Series	600		165	177	10.3354/meps12633
Gillis, AJ; Ceriani, SA; Seminoff, JA; Fuentes, MMPB	Foraging ecology and diet selection of juvenile green turtles in the Bahamas: insights from stable isotope analysis and prey mapping	2018	Marine Ecology Progress Series	599		225	238	10.3354/meps12635
Hays, GC; Alcoverro, T; Christianen, MJA; Duarte, CM; Hamann, M; Macreadie, PI; Marsh, HD; Rasheed, MA; Thums, M; Unsworth, RKF; York, PH; Esteban, N	New tools to identify the location of seagrass meadows: Marine grazers as habitat indicators	2018	Frontiers in Marine Science	4	FEB			10.3389/fmars.2018.00009
Scott, AL; York, PH; Duncan, C; Macreadie, PI; Connolly, RM; Ellis, MT; Jarvis, JC; Jinks, KI; Marsh, H; Rasheed, MA	The role of herbivory in structuring tropical seagrass ecosystem service delivery	2018	Frontiers in Plant Science	9				10.3389/fpls.2018.00127
Burgett, CM; Burkholder, DA; Coates, KA; Fourqurean, VL; Kenworthy, WJ; Manuel, SA; Outerbridge, ME; Fourqurean, JW	Ontogenetic diet shifts of green sea turtles (Chelonia mydas) in a mid-ocean developmental habitat	2018	Marine Biology	165	2			10.1007/s00227-018-3290-6
Pawlik, JR; Loh, T-L; McMurray, SE	A review of bottom-up vs. top-down control of sponges on Caribbean fore-reefs: What's old, what's new, and future directions	2018	PeerJ	2018	1			10.7717/peerj.4343

Authors	Title	Year	Source title	Volume	Issue	Page s	Page e	DOI
van Dijk, KJ; Bricker, E; van Tussenbroek, BI; Waycott, M	Range-wide population genetic structure of the Caribbean marine angiosperm Thalassia testudinum	2018	Ecology and Evolution	8	18	9478	9490	10.1002/ece3.4443
Alberti, M; Reich, S	A palaeoecological review of the lower Gatun Formation (Miocene) of Panama with special emphasis on trophic relationships	2018	Palaeobiodiversity and palaeoenvironments	98	4	571	591	10.1007/s12549-018-0326-3
Johnson, RA; Gulick, AG; Bolten, AB; Bjorndal, KA	Blue carbon stores in tropical seagrass meadows maintained under green turtle grazing	2017	Scientific Reports	7	1			10.1038/s41598-017-13142-4
Tol, SJ; Jarvis, JC; York, PH; Grech, A; Congdon, BC; Coles, RG	Long distance biotic dispersal of tropical seagrass seeds by marine mega-herbivores	2017	Scientific Reports	7	1			10.1038/s41598-017-04421-1
Bjorndal, KA; Bolten, AB; Chaloupka, M; Saba, VS; Bellini, C; Marcovaldi, MAG; Santos, AJB; Bortolon, LFW; Meylan, AB; Meylan, PA; Gray, J; Hardy, R; Brost, B; Bresette, M; Gorham, JC; Connett, S; Crouchley, BVS; Dawson, M; Hayes, D; Diez, CE; van Dam, RP; Willis, S; Nava, M; Hart, KM; Cherkiss, MS; Crowder, AG; Pollock, C; Hillis-Starr, Z; Muñoz Tenería, FA; Herrera-Pavón, R; Labrada-Martagón, V; Lorences, A; Negrete-Philippe, A; Lamont, MM; Foley, AM; Bailey, R; Carthy, RR; Scarpino, R; McMichael, E; Provancha, JA; Brooks, A; Jardim, A; López-Mendilaharsu, M; González-Paredes, D; Estrades, A; Fallabrino, A; Martínez-Souza, G; Vélez-Rubio, GM; Boulon, RH; Collazo, JA; Wershoven, R; Guzmán Hernández, V; Stringell, TB; Sanghera, A; Richardson, PB; Broderick, AC; Phillips, Q; [illegible]	Ecological regime shift drives declining growth rates of sea turtles throughout the West Atlantic	2017	Global Change Biology	23	11	4556	4568	10.1111/gcb.13712
van Tussenbroek, BI; Morales, LFG	Grazing by green sea-turtles does not affect reproductive fitness in Thalassia testudinum	2017	Aquatic Botany	141		10	16	10.1016/j.aquabot.2017.05.003
Christiansen, F; Esteban, N; Mortimer, JA; Dujon, AM; Hays, GC	Diel and seasonal patterns in activity and home range size of green turtles on their foraging grounds revealed by extended Fastloc-GPS tracking	2017	Marine Biology	164	1			10.1007/s00227-016-3048-y
Mutchler, T; Hoffman, DK	Response of seagrass (Thalassia testudinum) metrics to short-term nutrient enrichment and grazing manipulations	2017	Journal of Experimental Marine Biology and Ecology	486		105	113	10.1016/j.jembe.2016.09.015
Cuvillier, A; Villeneuve, N; Cordier, E; Kolasinski, J; Maurel, L; Farnier, N; Frouin, P	Causes of seasonal and decadal variability in a tropical seagrass seascape (Reunion Island, south western Indian Ocean)	2017	Estuarine Coastal and Shelf Science	184		90	101	10.1016/j.ecss.2016.10.046
Bessey, C; Heithaus, MR; Fourqurean, JW; Gastrich, KR; Burkholder, DA	Importance of teleost macrograzers to seagrass composition in a subtropical ecosystem with abundant populations of megagrazers and predators	2016	Marine Ecology Progress Series	553		81	92	10.3354/meps11790
Holzer, KK; McGlathery, KJ	Cultivation grazing response in seagrass may depend on phosphorus availability	2016	Marine Biology	163	4			10.1007/s00227-016-2855-5
Gama, LR; Domit, C; Broadhurst, MK; Fuentes, MMPB; Millar, RB	Green turtle Chelonia mydas foraging ecology at 25 degrees S in the western Atlantic: evidence to support a feeding model driven by intrinsic and extrinsic variability	2016	Marine Ecology Progress Series	542		209	219	10.3354/meps11576
Bakker, ES; Wood, KA; Pages, JF; Veen, GF; Christianen, MJA; Santamaria, L; Nolet, BA; Hilt, S	Herbivory on freshwater and marine macrophytes: A review and perspective	2016	Aquatic Botany	135		18	36	10.1016/j.aquabot.2016.04.008
Bessey, C; Heithaus, MR	Ecological niche of an abundant teleost Pelates octolineatus in a subtropical seagrass ecosystem	2015	Marine Ecology Progress Series	541		195	204	10.3354/meps11542
Chambault, P; Pinaud, D; Vantrepotte, V; Kelle, L; Entraygues, M; Guinet, C; Berzins, R; Bilo, K; Gaspar, P; De Thoisy, B; Maho, YL; Chevallier, D	Dispersal and diving adjustments of the green turtle Chelonia mydas in response to dynamic environmental conditions during post-nesting migration	2015	PLoS ONE	10	9			10.1371/journal.pone.0137340
Benga, G; Chapman, BE; Romeo, T; Cox, GC; Kuchel, PW	Morphology and water permeability of red blood cells from green sea turtle (Chelonia mydas)	2015	Protoplasma	252	4	1181	1185	10.1007/s00709-014-0747-4
Thomson, JA; Burkholder, DA; Heithaus, MR; Fourqurean, JW; Fraser, MW; Statton, J; Kendrick, GA	Extreme temperatures, foundation species, and abrupt ecosystem change: an example from an iconic seagrass ecosystem	2015	Global Change Biology	21	4	1463	1474	10.1111/gcb.12694
Heck, KL; Fodrie, FJ; Madsen, S; Baillie, CJ; Byron, DA	Seagrass consumption by native and a tropically associated fish species: Potential impacts of the tropicalization of the northern Gulf of Mexico	2015	Marine Ecology Progress Series	520		165	173	10.3354/meps11104
Hernández, ALM; Van Tussenbroek, BI	Patch dynamics and species shifts in seagrass communities under moderate and high grazing pressure by green sea turtles	2014	Marine Ecology Progress Series	517		143	157	10.3354/meps11068
Ebrahim, A; Olds, AD; Maxwell, PS; Pitt, KA; Burfeind, DD; Connolly, RM	Herbivory in a subtropical seagrass ecosystem: Separating the functional role of different grazers	2014	Marine Ecology Progress Series	511		83	91	10.3354/meps10901
Van Tussenbroek, BI; Cortés, J; Collin, R; Fonseca, AC; Gayle, PMH; Guzmán, HM; Jácome, GE; Juman, R; Koltes, KH; Oxenford, HA; Rodríguez-Ramirez, A; Samper-Villarreal, J; Smith, SR; Tschirky, JJ; Weil, E	Caribbean-wide, long-term study of seagrass beds reveals local variations, shifts in community structure and occasional collapse	2014	PLoS ONE	9	3			10.1371/journal.pone.0090600
Christianen, MJA; Herman, PMJ; Bouma, TJ; Lamers, LPM; Van Katwijk, MM; Van Der Heide, T; Mumby, PJ; Silliman, BR; Engelhard, SL; Van De Kerk, M; Kiswara, W; Van De Koppel, J	Habitat collapse due to overgrazing threatens turtle conservation in marine protected areas	2014	Proceedings of the Royal Society B: Biological Sciences	281	1777			10.1098/rspb.2013.2890
Heithaus, MR; Alcoverro, T; Arthur, R; Burkholder, DA; Coates, KA; Christianen, MJA; Kelkar, N; Manuel, SA; Wirsing, AJ; Kenworthy, WJ; Fourqurean, JW	Seagrasses in the age of sea turtle conservation and shark overfishing	2014	Frontiers in Marine Science	1	AUG			10.3389/fmars.2014.00028
Lacey, EA; Collado-Vides, L; Fourqurean, JW	Morphological and physiological responses of seagrasses (Alismatales) to grazers (Testudines: Cheloniidae) and the role of these responses as grazing patch abandonment cues [Respuestas morfológicas y fisiológicas de los pastos marinos (Alismatales) a los herbívoros (Testudines: Cheloniidae) y el papel de estas respuestas como señales de abandono de parches de pastoreo]	2014	Revista de Biologia Tropical	62	4	1535	1548	10.15517/rbt.v62i4.12844
Arthur, KE; Kelez, S; Larsen, T; Choy, CA; Popp, BN	Tracing the biosynthetic source of essential amino acids in marine turtles using δ13C fingerprints	2014	Ecology	95	5	1285	1293	10.1890/13-0263.1
Becking, LE; van Bussel, TCJM; Debrot, AO; Christianen, MJA	First record of a Caribbean green turtle (Chelonia mydas) grazing on invasive seagrass (Halophila stipulacea)	2014	Caribbean Journal of Marine Science	48	2-3	162	163	10.18475/cjos.v48i3.a05
Rossini, RA; Rueda, JL; Tibbetts, IR	Feeding ecology of the seagrass-grazing nerite Smaragdia souverbiana (Montrouzier, 1863) in subtropical seagrass beds of eastern Australia	2014	Journal of Molluscal science	80		139	147	10.1093/mollus/eyu003
Burkholder, DA; Heithaus, MR; Fourqurean, JW; Wirsing, A; Dill, LM	Patterns of top-down control in a seagrass ecosystem: Could a roving apex predator induce a behaviour-mediated trophic cascade?	2013	Journal of Animal Ecology	82	6	1192	1202	10.1111/1365-2656.12097
Arthur, R; Kelkar, N; Alcoverro, T; Madhusudan, MD	Complex ecological pathways underlie perceptions of conflict between green turtles and fishers in the Lakshadweep Islands	2013	Biological Conservation	167		25	34	10.1016/j.biocon.2013.07.014
Kelkar, N; Arthur, R; Marbà, N; Alcoverro, T	Greener pastures? High-density feeding aggregations of green turtles precipitate species shifts in seagrass meadows	2013	Journal of Ecology	101	5	1158	1168	10.1111/1365-2745.12122
Kelkar, N; Arthur, R; Marba, N; Alcoverro, T	Green turtle herbivory dominates the fate of seagrass primary production in the Lakshadweep islands (Indian Ocean)	2013	Marine Ecology Progress Series	485		235	243	10.3354/meps10406
Okuyama, J; Nakajima, K; Noda, T; Kimura, S; Kamihata, H; Kobayashi, M; Arai, N; Kagawa, S; Kawabata, Y; Yamada, H	Ethogram of Immature Green Turtles: Behavioral Strategies for Somatic Growth in Large Marine Herbivores	2013	PLoS ONE	8	6			10.1371/journal.pone.0065783
Christianen, MJA; van Belzen, J; Herman, PMJ; van Katwijk, MM; Lamers, LPM; van Leent, PJM; Bouma, TJ	Low-Canopy Seagrass Beds Still Provide Important Coastal Protection Services	2013	PLoS ONE	8	5			10.1371/journal.pone.0062413
Vander Zanden, HB; Arthur, KE; Bolten, AB; Popp, BN; Lagueux, CJ; Harrison, E; Campbell, CL; Bjorndal, KA	Trophic ecology of a green turtle breeding population	2013	Marine Ecology Progress Series	476		237	249	10.3354/meps10185
Burkepile, DE	Comparing aquatic and terrestrial grazing ecosystems: Is the grass really greener?	2013	Oikos	122	2	306	312	10.1111/j.1600-0706.2012.20716
Baggett, LP; Heck, KL; Frankovich, TA; Armitage, AR; Fourqurean, JW	Stoichiometry, growth, and fecundity responses to nutrient enrichment by invertebrate grazers in sub-tropical turtle grass (Thalassia testudinum) meadows	2013	Marine Biology	160	1	169	180	10.1007/s00227-012-2075-6
Chiu, SH; Huang, YH; Lin, HJ	Carbon budget of leaves of the tropical intertidal seagrass Thalassia hemprichii	2013	Estuarine Coastal and Shelf Science	125		27	35	10.1016/j.ecss.2013.03.026
Reisser, J; Proietti, M; Sazima, I; Kinas, P; Horta, P; Secchi, E	Feeding ecology of the green turtle (Chelonia mydas) at rocky reefs in western South Atlantic	2013	Marine Biology	160	12	3169	3179	10.1007/s00227-013-2304-7
Burkholder, DA; Heithaus, MR; Fourqurean, JW	Feeding preferences of herbivores in a relatively pristine subtropical seagrass ecosystem	2012	Marine and Freshwater Research	63	11	1051	1058	10.1071/MF12029
González Carman, V; Falabella, V; Maxwell, S; Albareda, D; Campagna, C; Mianzan, H	Revisiting the ontogenetic shift paradigm: The case of juvenile green turtles in the SW Atlantic	2012	Journal of Experimental Marine Biology and Ecology	429		64	72	10.1016/j.jembe.2012.06.007
Townsend, KA; Altvater, J; Thomas, MC; Schuyler, QA; Nette, GW	Death in the octopus' garden: Fatal blue-lined octopus envenomations of adult green sea turtles	2012	Marine Biology	159	3	689	695	10.1007/s00227-011-1846-9
Christianen, MJA; Govers, LL; Bouma, TJ; Kiswara, W; Roelofs, JG; Lamers, LPM; van Katwijk, MM	Marine megaherbivore grazing may increase seagrass tolerance to high nutrient loads	2012	Journal of Ecology	100	2	546	560	10.1111/j.1365-2745.2011.01900
Heithaus, MR; Wirsing, AJ; Dill, LM	The ecological importance of intact top-predator populations: a synthesis of 15 years of research in a seagrass ecosystem	2012	Marine and Freshwater Research	63	11	1039	1050	10.1071/MF12024

Authors	Title	Year	Source title	Volume	Issue	Page s	Page e	DOI
Marco-Mendez, C; Prado, P; Heck, KL; Cebrian, J; Sanchez-Lizaso, JL	Epiphytes mediate the trophic role of sea urchins in Thalassia testudinum seagrass beds	2012	Marine Ecology Progress Series	460		91	100	10.3354/meps09781
Burkholder, DA; Heithaus, MR; Thomson, JA; Fourqurean, JW	Diversity in trophic interactions of green sea turtles Chelonia mydas on a relatively pristine coastal foraging ground	2011	Marine Ecology Progress Series	439		277	293	10.3354/meps09313
Lemons, G; Lewison, R; Komoroske, L; Gaos, A; Lai, C-T; Dutton, P; Eguchi, T; LeRoux, R; Seminoff, JA	Trophic ecology of green sea turtles in a highly urbanized bay: Insights from stable isotopes and mixing models	2011	Journal of Experimental Marine Biology and Ecology	405	1-2	25	32	10.1016/j.jembe.2011.05.012
Russell, DJ; Hargrove, S; Balazs, GH	Marine sponges, other animal food, and nonfood items found in digestive tracts of the herbivorous marine turtle chelonia mydas in Hawai'i	2011	Pacific Science	65	3	375	381	10.2984/65.3.375
Santos, RG; Martins, AS; Farias, JD; Horta, PA; Pinheiro, HT; Torezani, E; Baptistotte, C; Seminoff, JA; Balazs, GH; Work, TM	Coastal habitat degradation and green sea turtle diets in Southeastern Brazil	2011	Marine Pollution Bulletin	62	6	1297	1302	10.1016/j.marpolbul.2011.03.004
Fourqurean, JW; Manuel, S; Coates, KA; Kenworthy, WJ; Smith, SR	Effects of excluding sea turtle herbivores from a seagrass bed: Overgrazing may have led to loss of seagrass meadows in Bermuda	2010	Marine Ecology Progress Series	419		223	232	10.3354/meps08853
Ballorain, K; Ciccione, S; Bourjea, J; Grizel, H; Enstipp, M; Georges, J-Y	Habitat use of a multispecific seagrass meadow by green turtles Chelonia mydas at Mayotte Island	2010	Marine Biology	157	12	2581	2590	10.1007/s00227-010-1520-7
Lal, A; Arthur, R; Marbà, N; Lill, AWT; Alcoverro, T	Implications of conserving an ecosystem modifier: Increasing green turtle (Chelonia mydas) densities substantially alters seagrass meadows	2010	Biological Conservation	143	11	2730	2738	10.1016/j.biocon.2010.07.020
Cardona, L; Campos, P; Levy, Y; Demetropoulos, A; Margaritoulis, D	Asynchrony between dietary and nutritional shifts during the ontogeny of green turtles (Chelonia mydas) in the Mediterranean	2010	Journal of Experimental Marine Biology and Ecology	393	1-2	83	89	10.1016/j.jembe.2010.07.004
Riosmena-Rodríguez, R; Talavera-Sáenz, A; Acosta-Vargas, B; Gardner, SC	Heavy metals dynamics in seaweeds and seagrasses in Bahía Magdalena, B.C.S., México	2010	Journal of Applied Phycology	22	3	283	291	10.1007/s10811-009-9457-2
Olsen, YS; Valiela, I	Effect of sediment nutrient enrichment and grazing on turtle grass Thalassia testudinum in Jobos Bay, Puerto Rico	2010	Estuaries and Coasts	33	3	769	783	10.1007/s12237-009-9256-7
Lazar, B; Zuljević, A; Holcer, D	Diet composition of a green turtle, chelonia mydas, from the adriatic sea	2010	Natura Croatica	19	1	263	271	
Armitage, AR; Fourqurean, JW	Stable isotopes reveal complex changes in trophic relationships following nutrient addition in a coastal marine ecosystem	2009	Estuaries and Coasts	32	6	1152	1164	10.1007/s12237-009-9219-z
Cardona, L; Aguilar, A; Pazos, L	Delayed ontogenic dietary shift and high levels of omnivory in green turtles (Chelonia mydas) from the NW coast of Africa	2009	Marine Biology	156	7	1487	1495	10.1007/s00227-009-1188-z
Herbert, DA; Fourqurean, JW	Phosphorus Availability and Salinity Control Productivity and Demography of the Seagrass Thalassia testudinum in Florida Bay	2009	Estuaries and Coasts	32	1	188	201	10.1007/s12237-008-9116-x
Sheppard, JK; Carter, AB; McKenzie, LJ; Pitcher, CR; Coles, RG	Spatial patterns of sub-tidal seagrasses and their tissue nutrients in the Torres Strait, northern Australia: Implications for management	2008	Continental Shelf Research	28	16	2282	2291	10.1016/j.csr.2008.03.033
Macías-Zamora, JV; Sánchez-Osorio, JL; Ríos-Mendoza, LM; Ramírez-Álvarez, N; Huerta-Díaz, MA; López-Sánchez, D	Trace metals in sediments and Zostera marina of San Ignacio and Ojo de Liebre lagoons in the central pacific coast of Baja California, Mexico	2008	Archives of Environmental Contamination and Toxicology	55	2	218	228	10.1007/s00244-007-9115-0
Takahashi, EM; Arthur, KE; Shaw, GR	Occurrence of okadaic acid in the feeding grounds of dugongs (Dugong dugon) and green turtles (Chelonia mydas) in Moreton Bay, Australia	2008	Harmful Algae	7	4	430	437	10.1016/j.hal.2007.09.003
Arthur, K; Limpus, C; Balazs, G; Capper, A; Udy, J; Shaw, G; Keuper-Bennett, U; Bennett, P	The exposure of green turtles (Chelonia mydas) to tumour promoting compounds produced by the cyanobacterium Lyngbya majuscula and their potential role in the aetiology of fibropapillomatosis	2008	Harmful Algae	7	1	114	125	10.1016/j.hal.2007.06.001
terHorst, CP; Munguia, P	Measuring ecosystem function: consequences arising from variation in biomass-productivity relationships	2008	Community Ecology	9	1	39	44	10.1556/ComEc.9.2008.1.5
Marsh, H; Kwan, D	Temporal variability in the life history and reproductive biology of female dugongs in Torres Strait: The likely role of sea grass dieback	2008	Continental Shelf Research	28	16	2152	2159	10.1016/j.csr.2008.03.023
Bologna, PAX; Papagian, R; Regetz, S; Dale, C	Assessment of turtle grass (Thalassia testudinum ex Banks Konig) community structure in a UNESCO Biosphere Reserve	2008	Journal of Experimental Marine Biology and Ecology	365	2	148	155	10.1016/j.jembe.2008.08.011
Arthur, KE; O'neil, JM; Limpus, CJ; Abernathy, K; Marshall, G	Using animal-borne imaging to assess green turtle (Chelonia mydas) foraging ecology in Moreton Bay, Australia	2007	Marine Technology Society Journal	41	4	9	13	10.4031/002533207787441953
Short, F; Carruthers, T; Dennison, W; Waycott, M	Global seagrass distribution and diversity: A bioregional model	2007	Journal of Experimental Marine Biology and Ecology	350	1-2	3	20	10.1016/j.jembe.2007.06.012
Heithaus, MR; Frid, A; Wirsing, AJ; Dill, LM; Fourqurean, JW; Burkholder, D; Thomson, J; Bejder, L	State-dependent risk-taking by green sea turtles mediates top-down effects of tiger shark intimidation in a marine ecosystem	2007	Journal of Animal Ecology	76	5	837	844	10.1111/j.1365-2656.2007.01260
Murdoch, TJT; Glasspool, AF; Outerbridge, M; Ward, J; Manuel, S; Gray, J; Nash, A; Coates, KA; Pitt, J; Fourqurean, JW; Barnes, PA; Vierros, M; Holzer, K; Smith, SR	Large-scale decline in offshore seagrass meadows in Bermuda	2007	Marine Ecology Progress Series	339		123	130	10.3354/meps339123
Kuiper-Linley, M; Johnson, CR; Lanyon, JM	Effects of simulated green turtle regrazing on seagrass abundance, growth and nutritional status in Moreton Bay, south-east Queensland, Australia	2007	Marine and Freshwater Research	58	5	492	503	10.1071/MF06241
Moran, KL; Bjorndal, KA	Simulated green turtle grazing affects nutrient composition of the seagrass Thalassia testudinum	2007	Marine Biology	150	6	1083	1092	10.1007/s00227-006-0427-9
Aragones, LV; Lawler, IR; Foley, WJ; Marsh, H	Dugong grazing and turtle cropping: Grazing optimization in tropical seagrass systems?	2006	Oecologia	149	4	635	647	10.1007/s00442-006-0477-1
Gil, M; Armitage, AR; Fourqurean, JW	Nutrient impacts on epifaunal density and species composition in a subtropical seagrass bed	2006	Hydrobiologia	569	1	437	447	10.1007/s10750-006-0147-7
Heck Jr; KL; Valentine, JF	Plant-herbivore interactions in seagrass meadows	2006	Journal of Experimental Marine Biology and Ecology	330	1	420	436	10.1016/j.jembe.2005.12.044
Çelik, A; Kaska, Y; Bağ, H; Aureggi, M; Semiz, G; Kartal, AA; Elçi, L	Heavy metal monitoring around the nesting environment of green sea turtles in Turkey	2006	Water, Air, and Soil Pollution	169	1-4	67	79	10.1007/s11270-006-1562-0
Moran, KL; Bjorndal, KA	Simulated green turtle grazing affects structure and productivity of seagrass pastures	2005	Marine Ecology Progress Series	305		235	247	10.3354/meps305235
André, J; Gyuris, E; Lawler, IR	Comparison of the diets of sympatric dugongs and green turtles on the Orman Reefs, Torres Strait, Australia	2005	Wildlife Research	32	1	53	62	10.1071/WR04015
Waycott, M; Longstaff, BJ; Mellors, J	Seagrass population dynamics and water quality in the Great Barrier Reef region: A review and future research directions	2005	Marine Pollution Bulletin	51	1-4	343	350	10.1016/j.marpolbul.2005.01.017
Hays, CG	Effect of nutrient availability, grazer assemblage and seagrass source population on the interaction between Thalassia testudinum (turtle grass) and its algal epiphytes	2005	Journal of Experimental Marine Biology and Ecology	314	1	53	68	10.1016/j.jembe.2004.08.017
Armitage, AR; Frankovich, TA; Heck, KL; Fourqurean, JW	Experimental nutrient enrichment causes complex changes in seagrass, microalgae, and macroalgae community structure in Florida Bay	2005	Estuaries	28	3	422	434	10.1007/BF02693924
Bewles, JW; Bell, SS	Simulated herbivory and the dynamics of disease in Thalassia testudinum	2004	Marine Ecology Progress Series	283		127	132	10.3354/meps283127
Hauxwell, J; Osenberg, CW; Frazer, TK	Conflicting management goals: Manatees and invasive competitors inhibit restoration of a native macrophyte	2004	Ecological Applications	14	2	571	586	10.1890/02-5250
Russell, DJ; Balazs, GH; Phillips, RC; Kam, AKH	Discovery of the sea grass Halophila decipiens (Hydrocharitaceae) in the diet of the Hawaiian green turtle, Chelonia mydas	2003	Pacific Science	57	4	393	397	10.1353/psc.2003.0034
Hays, GC; Glen, F; Broderick, AC; Godley, BJ; Metcalfe, JD	Behavioural plasticity in a large marine herbivore: Contrasting patterns of depth utilisation between two green turtle (Chelonia mydas) populations	2002	Marine Biology	141	5	985	990	10.1007/s00227-002-0885-7
Carruthers, TJB; Dennison, WC; Longstaff, BJ; Waycott, M; Abal, EG; McKenzie, LJ; Lee Long, WJ	Seagrass habitats of northeast Australia: Models of key processes and controls	2002	Bulletin of Marine Science	71	3	1153	1169	
Kirsch, KD; Valentine, JF; Heck Jr; KL	Parrotfish grazing on turtlegrass Thalassia testudinum: Evidence for the importance of seagrass consumption in food web dynamics of the Florida Keys National Marine Sanctuary	2002	Marine Ecology Progress Series	227		71	85	10.3354/meps227071
Heithaus, MR	The biology of tiger sharks, Galeocerdo cuvier, in Shark Bay, Western Australia: Sex ratio, size distribution, diet, and seasonal changes in catch rates	2001	Environmental Biology of Fishes	61	1	25	36	10.1023/A:1011021210685
Valentine, JF; Heck Jr; KL	The role of leaf nitrogen content in determining turtlegrass (Thalassia testudinum) grazing by a generalized herbivore in the northeastern Gulf of Mexico	2001	Journal of Experimental Marine Biology and Ecology	258	1	65	86	10.1016/S0022-0981(00)00342-7
Bjorndal, KA; Bolten, AB; Chaloupka, MY	Green turtle somatic growth model: Evidence fordensity dependence	2000	Ecological Applications	10	1	269	282	10.1890/1051-0761(2000)010[02[illegible]
Zann, LP	Northeastern Australia: The Great Barrier Reef region	2000	Seas at the millennium - an environmental evaluation - Volume 2			611	628	
Aragones, L; Marsh, H	Impact of Dugong grazing and turtle cropping on tropical seagrass communities	2000	Pacific Conservation Biology	5	4	286	288	
Valentine, JF; Heck, KL; Busby Jr; J; Webb, D	Experimental evidence that herbivory increases shoot density and productivity in a subtropical turtlegrass (Thalassia testudinum) meadow	1997	Oecologia	112	2	193	200	10.1007/s004420050300
Jackson, JBC	Reefs since Columbus	1997	Coral Reefs	16	SUPPL	S23	S32	10.1007/s003380050238
Jupp, BP; Durako, MJ; Kenworthy, WJ; Thayer, GW; Schillak, L	Distribution, abundance, and species composition of seagrasses at several sites in Oman	1996	Aquatic Botany	53	3-4	199	213	10.1016/0304-3770(96)01023-6
Williams, SL	Thalassia testudinum productivity and grazing by green turtles in a highly disturbed seagrass bed	1988	Marine Biology	98	3	447	455	10.1007/BF00391121
Garnett, ST; Price, IR; Scott, FJ	The diet of the green turtle, chelonia mydas (L.), in torres strait	1985	Wildlife Research	12	1	103	112	10.1071/WR9850103
Thayer, GW; Bjorndal, KA; Ogden, JC; Willia, SL; Zieman, JC	Role of larger herbvores in seagrass communities	1984	Estuaries	7	4	351	376	10.2307/1351619
Tribble, GW	Reef-basel herbivores and the distribution of two seagrasses (Syringodium filiforme and Thalassia testudinum) in the San Blas Islands (Western Caribbean)	1981	Marine Biology	65	3	277	281	10.1007/BF00397122
Bjorndal, KA	Nutrition and grazing behavior of the green turtle Chelonia mydas	1980	Marine Biology	56	2	147	154	10.1007/BF00397131
Johnstone, IM	The ecology and distribution of Papua New Guinea seagrasses. II. The Fly Islands and Raboin Island	1978	Aquatic Botany	5	C	235	243	10.1016/0304-3770(78)90066-9
